# Supplementary material for: Rates of knee arthroplasty in patients with a history of arthroscopic chondroplasty: results from a retrospective cohort study utilising the National Hospital Episode Statistics for England
Source: BMJ Open. 2020 Apr 16;10(4):e030609. doi: 10.1136/bmjopen-2019-030609 (PMC7200031; doi:10.1136/bmjopen-2019-030609)
Supplement: Supplementary data [file bmjopen-2019-030609supp001.pdf]

Appendix 1: OPCS procedure code list

PROCEDURE	OPCS 4.4	OPCS 4.5	OPCS 4.6	OPCS 4.7	Description
Chondroplasty*	W83.3*	W83.3*	W83.3*	W83.3*	Endoscopic shaving of articular cartilage
Chondroplasty*	W83.4*	W83.4*	W83.4*	W83.4*	Endoscopic articular abrasion chondroplasty
Chondroplasty*	W83.5*	W83.5*	W83.5*	W83.5*	Endoscopic articular thermal chondroplasty
Chondroplasty*	W83.6*	W83.6*	W83.6*	W83.6*	Endoscopic excision of articular cartilage NEC
Chondroplasty*	W89.1*	W89.1*	W89.1*	W89.1*	Endoscopic chondroplasty NEC
Microfracture*	W83.1*	W83.1*	W83.1*	W83.1*	Endoscopic drilling of lesion of articular cartilage
Microfracture*	W84.5*	W84.5*	W84.5*	W84.5*	Endoscopic drilling of epiphysis for repair of articular cartilage
Meniscal surgery	W82.2	W82.2	W82.2	W82.2	Endoscopic resection of semilunar cartilage NEC
Meniscal surgery	W82.3	W82.3	W82.3	W82.3	Endoscopic repair of semilunar cartilage
Meniscal surgery	W82.1	W82.1	W82.1	W82.1	Endoscopic total excision of semilunar cartilage
Ligament reconstruction (exclusion)*	W74.2	W74.2	W74.2	W74.2	Reconstruction of intra-articular ligament NEC
Ligament reconstruction (exclusion)*	W84.1	W84.1	W84.1	W84.1	Endoscopic repair of intra-articular ligament
Ligament reconstruction (exclusion)*	W84.2	W84.2	W84.2	W84.2	Endoscopic reattachment of intra-articular ligament
Ligament reconstruction (exclusion)*	W72.3	W72.3	W72.3	W72.3	Primary prosthetic replacement of intra-articular ligament
Ligament reconstruction (exclusion)*	W72.4	W72.4	W72.4	W72.4	Prosthetic replacement of intra-articular ligament NEC
Arthroplasty*	W43.1	W43.1	W43.1	W43.1	Primary total prosthetic replacement of joint using cement NEC
Arthroplasty*	W44.1	W44.1	W44.1	W44.1	Primary total prosthetic replacement of joint not using cement NEC
Arthroplasty*	W44.8	W44.8	W44.8	W44.8	Other specified total prosthetic replacement of other joint not using cement
Arthroplasty*	W44.9	W44.9	W44.9	W44.9	Unspecified total prosthetic replacement of other joint not using cement
Arthroplasty*	W45.1	W45.1	W45.1	W45.1	Primary total prosthetic replacement of joint NEC
Arthroplasty*	W45.8	W45.8	W45.8	W45.8	Other specified other total prosthetic replacement of other joint
Arthroplasty*	W45.9	W45.9	W45.9	W45.9	Unspecified other total prosthetic replacement of other joint
Arthroplasty*	W53.8	W53.8	W53.8	W53.8	Other specified prosthetic replacement of articulation of other bone not using cement
Arthroplasty	W40.1	O18.1	O18.1	O18.1	Primary hybrid prosthetic replacement of knee joint using cement
Arthroplasty	W40.8	O18.8	O18.8	O18.8	Other specified hybrid prosthetic replacement of knee joint using cement
Arthroplasty	W40.9	O18.9	O18.9	O18.9	Unspecified hybrid prosthetic replacement of knee joint using cement
Arthroplasty	W40.1	W40.1	W40.1	W40.1	Primary total prosthetic replacement of knee joint using cement
Arthroplasty	W40.8	W40.8	W40.8	W40.8	Other specified total prosthetic replacement of knee joint using cement
Arthroplasty	W40.9	W40.9	W40.9	W40.9	Unspecified total prosthetic replacement of knee joint using cement
Arthroplasty	W41.1	W41.1	W41.1	W41.1	Primary total prosthetic replacement of knee joint not using cement
Arthroplasty	W41.8	W41.8	W41.8	W41.8	Other specified total prosthetic replacement of knee joint not using cement
Arthroplasty	W41.9	W41.9	W41.9	W41.9	Unspecified total prosthetic replacement of knee joint not using cement
Arthroplasty	W42.1	W42.1	W42.1	W42.1	Primary total prosthetic replacement of knee joint NEC
Arthroplasty	W42.8	W42.8	W42.8	W42.8	Other specified other total prosthetic replacement of knee joint
Arthroplasty	W42.9	W42.9	W42.9	W42.9	Unspecified other total prosthetic replacement of knee joint
Arthroplasty*	W58.1	W58.1	W58.1	W58.1	Primary resurfacing arthroplasty of joint

**: Additional site-specific knee code required (Z846, Z765, Z845, Z844, Z774, or Z787)*
