# Supplementary figures and images for: Rates of knee arthroplasty in patients with a history of arthroscopic chondroplasty: results from a retrospective cohort study utilising the National Hospital Episode Statistics for England
Source: BMJ Open. 2020 Apr 16;10(4):e030609. doi: 10.1136/bmjopen-2019-030609 (PMC7200031; doi:10.1136/bmjopen-2019-030609)

**Appendix 2:** Flow chart illustrating the extraction of the knee arthroplasty cohort

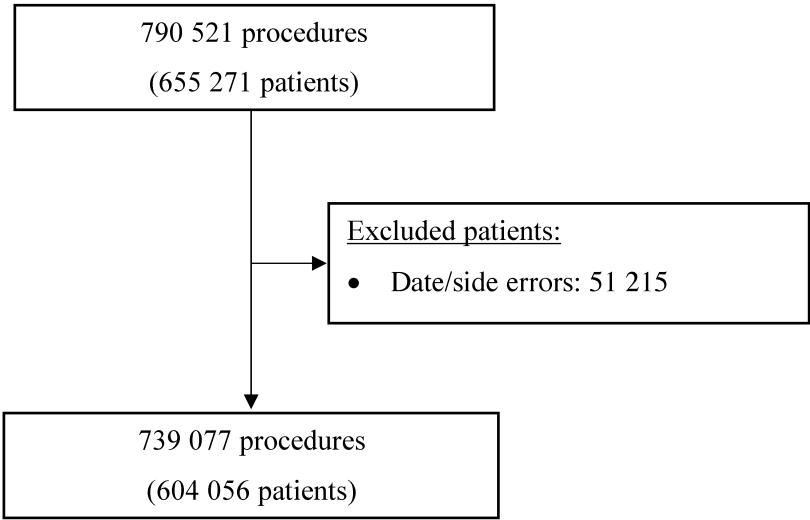

Supplement: Supplementary data [file bmjopen-2019-030609supp002.pdf]
