# Supplementary material for: Rates of knee arthroplasty in patients with a history of arthroscopic chondroplasty: results from a retrospective cohort study utilising the National Hospital Episode Statistics for England
Source: BMJ Open. 2020 Apr 16;10(4):e030609. doi: 10.1136/bmjopen-2019-030609 (PMC7200031; doi:10.1136/bmjopen-2019-030609)
Supplement: Author's manuscript [file bmjopen-2019-030609.draft_revisions.pdf]

BMJ Open is committed to open peer review. As part of this commitment we make the peer review history of every article we publish publicly available.

When an article is published we post the peer reviewers' comments and the authors' responses online. We also post the versions of the paper that were used during peer review. These are the versions that the peer review comments apply to.

The versions of the paper that follow are the versions that were submitted during the peer review process. They are not the versions of record or the final published versions. They should not be cited or distributed as the published version of this manuscript.

BMJ Open is an open access journal and the full, final, typeset and author-corrected version of record of the manuscript is available on our site with no access controls, subscription charges or pay-per-view fees (http://bmjopen.bmj.com).

If you have any questions on BMJ Open's open peer review process please email info.bmjopen@bmj.com

BMJ Open

Rates of knee arthroplasty in patients with a history of arthroscopic chondroplasty: results from a retrospective cohort study utilising the National Hospital Episode Statistics for England

Journal:	*BMJ Open*
Manuscript ID	bmjopen-2019-030609
Article Type:	Research
Date Submitted by the Author:	22-Mar-2019

Complete List of Authors:	Abram, Simon; University of Oxford Nuffield Department of Orthopaedics Rheumatology and Musculoskeletal Sciences,<br>Palmer, Antony; University of Oxford, Nuffield Department of Orthopaedics, Rheumatology and Musculoskeletal Sciences<br>Judge, Andrew; University of Bristol, Musculoskeletal Research Unit, Translational Health Sciences, Bristol Medical School, University of Bristol, Learning and Research Building, Level 1, Southmead Hospital, Southmead, BS10 5NB<br>Beard, David; University of Oxford, Nuffield Dept of Orthopaedics, Rheumatology and Musculoskeletal Sciences<br>Price, Andrew; University of Oxford, Nuffield Department of Orthopaedics, Rheumatology and Musculoskeletal Sciences

Keywords:	Knee < ORTHOPAEDIC & TRAUMA SURGERY, arthroscopy, chondroplasty, knee replacement, arthroplasty, osteoarthritis

SCHOLARONE™
Manuscripts

I, the Submitting Author has the right to grant and does grant on behalf of all authors of the Work (as defined in the below author licence), an exclusive licence and/or a non-exclusive licence for contributions from authors who are: i) UK Crown employees; ii) where BMJ has agreed a CC-BY licence shall apply, and/or iii) in accordance with the terms applicable for US Federal Government officers or employees acting as part of their official duties; on a worldwide, perpetual, irrevocable, royalty-free basis to BMJ Publishing Group Ltd ("BMJ") its licensees and where the relevant Journal is co-owned by BMJ to the co-owners of the Journal, to publish the Work in this journal and any other BMJ products and to exploit all rights, as set out in our licence.

The Submitting Author accepts and understands that any supply made under these terms is made by BMJ to the Submitting Author unless you are acting as an employee on behalf of your employer or a postgraduate student of an affiliated institution which is paying any applicable article publishing charge ("APC") for Open Access articles. Where the Submitting Author wishes to make the Work available on an Open Access basis (and intends to pay the relevant APC), the terms of reuse of such Open Access shall be governed by a Creative Commons licence – details of these licences and which Creative Commons licence will apply to this Work are set out in our licence referred to above.

Other than as permitted in any relevant BMJ Author's Self Archiving Policies, I confirm this Work has not been accepted for publication elsewhere, is not being considered for publication elsewhere and does not duplicate material already published. I confirm all authors consent to publication of this Work and authorise the granting of this licence.

For peer review only

Rates of knee arthroplasty in patients with a history of arthroscopic chondroplasty: results from a retrospective cohort study utilising the National Hospital Episode Statistics for England

1. S.G.F. Abram, MRCS[1,2]
2. A.J.R. Palmer, DPhil[1,2]
3. A. Judge, PhD[1,3,4]
4. D.J. Beard, DPhil[1,2]
5. A.J. Price, DPhil[1,2]

Author affiliations:

[1]Nuffield Department of Orthopaedics, Rheumatology and Musculoskeletal Sciences, University of Oxford
[2]NIHR Biomedical Research Centre, Oxford
[3]Musculoskeletal Research Unit, University of Bristol
[4]NIHR Biomedical Research Centre, Bristol

Author titles:

SGFA: NIHR Doctoral Research Fellow
AJRP: NIHR Clinical Lecturer
AJ: Professor of Translational Statistics
DJB: Professor of Musculoskeletal Sciences
AJP: Professor of Orthopaedics

Corresponding author:

Simon Abram

E: simon.abram@ndorms.ox.ac.uk

T: 01865 223425

Botnar Research Centre, University of Oxford

Nuffield Department of Orthopaedics, Rheumatology and Musculoskeletal Sciences

Windmill Road, Oxford OX3 7LD. UK

Keywords

knee; arthroscopy; chondroplasty; knee replacement; arthroplasty; outcomes; osteoarthritis

Word count

2487

For peer review only

ABSTRACT

OBJECTIVE

The purpose of this study was to analyse the rate of knee arthroplasty in the population of patients with a history of arthroscopic chondroplasty of the knee, in England, over ten years, with comparison to general population data for patients without a history of chondroplasty.

DESIGN

Retrospective cohort study

SETTING

English hospital episode statistics (HES) data

PARTICIPANTS AND INTERVENTIONS

Patients undergoing arthroscopic chondroplasty in England between 2007/08 and 2016/17 were identified. Patients undergoing previous arthroscopic knee surgery or simultaneous cruciate ligament reconstruction or microfracture in the same knee were excluded.

OUTCOMES

Patients subsequently undergoing a knee arthroplasty in the same knee were identified and mortality-adjusted survival analysis was performed (survival without undergoing knee arthroplasty). A cox proportional hazards model was used to identify factors associated with knee arthroplasty. Relative risk of knee arthroplasty (total or partial) in comparison to the general population was determined.

RESULTS

Through 2007-17, 157,730 eligible chondroplasty patients were identified. Within one-year, 5.91% (7984/135197; 95% CI 5.78 to 6.03) underwent knee arthroplasty and 14.22% (8145/57267; 95% CI 13.94 to 14.51) within five-years. Patients aged over 30 years with a history of chondroplasty were 17.32 times (risk ratio; 95% CI 16.81 to 17.84) more likely to undergo arthroplasty than the general population without a history of chondroplasty.

CONCLUSIONS

Patients with cartilage lesions of the knee, treated with arthroscopic chondroplasty, are at greater risk of subsequent knee arthroplasty than the general population and for a proportion of patients, there is insufficient benefit to prevent the need for knee arthroplasty within one- to five-years. These important new data will inform patients of the anticipated outcomes following this procedure. The risk in comparison to non-operative treatment remains unknown and there is an urgent need for a randomised clinical trial in this population.

For peer review only

STRENGTHS AND LIMITATIONS OF THIS STUDY

- Strengths of the data source analysed in this study include comprehensive, national, data collection and the ability to match treatment with outcomes, including by the laterality of intervention, over time.
- This is the largest cohort of patients undergoing arthroscopic chondroplasty that has been reported, with strict inclusion criteria excluding patients with a history of previous surgery to the same knee and those undergoing simultaneous ligament reconstruction or microfracture.
- All studies of this design rely on coding accuracy and some coding errors are inevitable and although outcomes were stratified by a range of patient factors, unmeasured potential confounders include body mass index, limb alignment, baseline radiographic status.
- Knee arthroplasty is an end-stage outcome and will underestimate the true burden and severity of symptomatic osteoarthritis in this population.
- The outcome had these patients not undergone arthroscopic chondroplasty remains unknown.

For peer review only

INTRODUCTION

Around 2 million knee arthroscopy procedures are performed worldwide each year.[1] Historically, knee washout and 'debridement' was shown to be ineffective for the treatment of advanced osteoarthritis.[2–4] For early osteoarthritis, however, a number of surgical and non-surgical treatments are available and treatment selection is challenging.[5] The aim of treatment in these cases is to improve symptoms and delay or prevent progressive osteoarthritis.[6]

Chondroplasty is a non-specific term that encompasses several techniques for the treatment of cartilage defects.[7] It includes debridement and abrasion using mechanical 'shavers' and, more recently, thermal or radiofrequency techniques have also emerged despite some concerns these techniques might risk inducing localised chondrocyte death.[7–11] Recent national guidance was cautiously supportive of radiofrequency chondroplasty for the treatment of "discrete chondral defects" based on a small number of clinical trials comparing the outcomes of mechanical and radiofrequency techniques.[12] It is not known which patients are most likely to benefit from chondroplasty procedures and when the procedure does not provide sustained benefit, knee arthroplasty is often indicated.

The purpose of this study was to determine the proportion of patients undergoing knee chondroplasty procedures that subsequently receive a knee arthroplasty in the same knee, with specific focus on the proportion of patients undergoing early arthroplasty with one-year or two-years of chondroplasty. Factors associated with the risk of subsequent arthroplasty are reported and the relative risk in comparison to the general population determined.

For peer review only

METHODS

Data source

National Hospital Episode Statistics (HES) data was obtained (application DARS-NIC-68703). HES contains a record of all patient attendances at National Health Service (NHS) hospitals in England.[13] The data is submitted by hospitals to claim payment for the services they provide and is also intended for secondary use, including research. HES includes episodes of care delivered in treatment centres (including those in the independent sector) funded by the NHS, episodes of care in England where patients are resident outside of England, and privately funded patients treated within NHS England hospitals. The information recorded in the HES database includes patient demographic and residence data, primary and secondary diagnoses including comorbidities, and all procedures undertaken.

Procedures

All HES records between 1 April 2007 and 31 March 2017 were extracted for patients undergoing arthroscopic chondroplasty. Patients undergoing previous arthroscopic knee surgery or simultaneous cruciate ligament reconstruction or microfracture in the same knee were excluded. Procedures were identified using the Classification of Surgical Operations and Procedures (OPCS-4) codes recorded within the HES data (see appendix 1 for OPCS-4 code list).[14] All knee arthroplasty (partial or total) procedures were also identified (appendix 2) for the whole population to enable the relative risk of knee arthroplasty with and without a history of chondroplasty to be determined.

Outcomes

The primary outcome was knee arthroplasty, matched to the side of any previous chondroplasty (using recorded OPCS-4 laterality codes).

Statistical analysis

Stata v15.1 (StataCorp, College Station, Texas, USA) was used to perform all analysis. In accordance with Office for National Statistics (ONS) and NHS Digital guidance, rates where the number of events was less than six were suppressed.[15] Procedures with date errors or missing laterality were excluded. The absolute rate of knee arthroplasty was determined at 1 year, 2 years, 5 years, and 8 years following arthroscopic chondroplasty as the proportion of the cohort with this minimum period of follow-up. Mortality adjusted Kaplan-Meier survival analysis (survival was defined as not undergoing knee arthroplasty) was also performed and stratified by patient age group and sex.

A Cox proportional-hazards model was used first to calculate the unadjusted hazard ratio of knee arthroplasty over time by age group, sex, index of multiple deprivation (quintile derived from regional factors in England including average income, employment, education, housing, and crime; 1=least deprived

For peer review only

area, 5=most deprived), ethnicity, modified Charlson comorbidity index (derived with maximum 5-year diagnosis code lookback period),[16–18] year of treatment (chondroplasty), rurality, and ethnicity respectively.[16–19] The hazard ratios were then adjusted including all these variables in the model.

The relative risk (risk ratio) of knee arthroplasty in the population of patients with a history of chondroplasty in comparison to the general population (without a history of chondroplasty) was estimated for the year 2016-17. All patients undergoing knee arthroplasty in 2016-17 were identified and the number of these patients with a recorded previous chondroplasty (in the prior 10-years of HES data), versus those without, made up the numerator for each respective population. The chondroplasty population denominator was the number of patients with a history of chondroplasty that had not undergone a knee arthroplasty prior to 2016-17. The denominator for the non-chondroplasty population was the ONS mid-year population estimate less the chondroplasty population.

Patient and public involvement

There was no patient and public involvement in this study.

For peer review only

RESULTS

Over the study period, 157,830 chondroplasty patients were identified as eligible for analysis (Figure 1). The mean age of the chondroplasty cohort was 51.7 year (SD 13.8) and 48.1% were female (Table 1).

Overall, following chondroplasty, 5.91% (7984/135197; 95% confidence interval [CI] 5.78 to 6.03) patients underwent knee arthroplasty within 1 year, 9.41% (10787/114592; 95% CI 9.24 to 9.58) within 2 years, 14.22% (8145/57267; 95% CI 13.94 to 14.51) within 5 years, and 17.61% (2879/16347; 95% CI 17.03 to 18.20) within 8 years (Table 2). The risk of arthroplasty was greater in female patients (adjusted hazard ratio [HR] 1.38; 95% CI 1.34 to 1.42) and in older patients (adjusted HR 1.33 per five years; 95% CI 1.32 to 1.34) (Table 3, Figure 2; Figure 3). Patients with a greater comorbidity index were also at increased risk of subsequently undergoing arthroplasty (adjusted HR 1.03 per five units Charlson index; 95% CI 1.01 to 1.05).

The risk of knee arthroplasty after chondroplasty fell slightly over time, by year of chondroplasty treatment (adjusted HR 0.95 per five years; 95% CI 0.92 to 0.98). Patients in regions of increased deprivation and patients of white ethnicity were at greater risk of subsequent arthroplasty (Table 2). Patients undergoing concurrent meniscal surgery were also at greater risk of subsequent arthroplasty (adjusted HR 1.09; 95% CI 1.06 to 1.13).

In 2016-17, the rate of knee arthroplasty was 3.49% (95% CI 3.39 to 3.60) in patients (aged 30 or older) with a recorded history of chondroplasty and 0.20% (95% CI 0.19 to 0.20) in patients without a record of chondroplasty. This corresponded to an overall relative risk of knee arthroplasty for the chondroplasty cohort patients of 17.32 times (risk ratio [RR]; 95% CI 16.81 to 17.84) that of the general population (Table 3).

Although the absolute annual rate of knee arthroplasty was low, the relative risk of undergoing knee arthroplasty at a younger age was greatly elevated in comparison to arthroplasty at an older age, as shown in Table 3. Patients aged 30-39 with a history of a previous chondroplasty were 170.92 times (RR; 95% CI 116.72 to 250.30) more likely to undergo knee arthroplasty than the general population, per year, in comparison to 11.09 times (RR; 95% CI 10.42 to 11.80) more likely for the over 69 age group.

For peer review only

DISCUSSION

Principal findings

Patients undergoing chondroplasty procedures of the knee have a 17 times increased risk of receiving a knee arthroplasty compared with the general population. Nearly 10% of patients will have received a knee arthroplasty within two years of the chondroplasty procedure. Young patients are at particularly elevated risk, reaching 171 times greater than the general population between 30 and 39 years of age. For a proportion of patients, the results indicate insufficient benefit to prevent the need for knee arthroplasty within one or two years.

Comparison to other studies

We previously reported trends in chondroplasty surgery in England, but data from other countries is not available.[20] The age-sex standardised rate of chondroplasty increased 191% from 17.6/100,000 (95% CI 17.2 to 18.0) in 2007/08 to 51.2/100,000 (95% CI 50.6 to 51.7) in 2016/17.[20] The rate of chondroplasty was greatest in patients aged 40-59 years (increasing 210% from 34.3/100,000 in 2007/08 to 106.4/100,000 in 2016/17.[20]

In England, although national guidance has been cautiously supportive of radiofrequency chondroplasty for specific indications. There is only limited evidence demonstrating the effectiveness of chondroplasty compared with alternative surgical or non-surgical treatments.[12] The only randomised studies have been limited to comparisons of different chondroplasty techniques.[10,12] Long-term outcomes following chondroplasty have yet to be reported.[12]

Older patients are much more likely to have generalised osteoarthritis, rather than “discrete chondral defects” for which the national guidance supports radiofrequency chondroplasty.[12,21] For more generalised osteoarthritis, chondroplasty is analogous to debridement and washout, where multiple clinical trials demonstrate no benefit.[2–4] The use of chondroplasty in the treatment of patients with more generalised chondral pathology is therefore unproven and not recommended.[22] In our study, there was considerable age-group variation in outcomes, with 18.8% of patients aged 60-79-years undergoing arthroplasty within two-years of chondroplasty, in comparison to 0.43% for patients undergoing chondroplasty aged 20-39-years. This observation is consistent with the presence of more established osteoarthritis in older age groups.

Our study represents a high-risk cohort of patients with cartilage damage. It is unknown from this observational data whether undergoing the chondroplasty procedure was beneficial to the symptoms or prognosis of these individuals over the full study period. Patients undergoing arthroplasty within 1-year of their arthroscopic chondroplasty, however, suggests sub-optimal treatment selection. These individuals are

For peer review only

highly unlikely to have had only localised or partial thickness lesions and our results may indicate that knee arthroplasty may have been a more appropriate treatment. It is unlikely that chondroplasty in these patients was a cost-effective choice of intervention, but whether chondroplasty can be cost-effective with optimal patient selection is unknown and requires evaluation in a high-quality randomised controlled trial.

Female patients were observed to be of greater risk of subsequent arthroplasty in our study. This has previously been observed following knee arthroscopy in the United States.[23] Patients of white ethnicity and greater deprivation were also at greater risk in our cohort. These findings may reflect differences in healthcare access including treatment thresholds for either the chondroplasty or knee arthroplasty, or differences in care seeking behaviour which has been shown to be influenced by socioeconomic, cultural, occupational, and psychological factors, or there could be biological factors underlying the observation.[24–26] Patients with a greater comorbidity index were more likely to undergo subsequent arthroplasty, and the reason for this is unclear. One possible explanation might be an association between comorbidity and higher body mass index (BMI), which is not recorded in this dataset, with patients having a greater BMI being more likely to progress to end-stage osteoarthritis, or that these patients had more severe pathology at the time of their index chondroplasty.[27] Patients undergoing concurrent meniscal surgery were also likely to undergo subsequent arthroplasty, which is expected given the association between meniscal injury, osteoarthritis, and knee arthroplasty.[28]

Recently, there has been renewed focus on the importance of and requirements for individualised patient consent.[29] Our findings make an important contribution to the current evidence, and patients can now be appropriately counselled and consented with knowledge of anticipated long-term outcomes.

<u>Strength and limitations</u>

A key strength of our study is the identification of all knee chondroplasty procedures performed in the National Health Service over a 10-year period, creating the largest reported cohort of patients receiving this procedure. Patients with a history of prior arthroscopy in the same knee, simultaneous ligament reconstruction or microfracture were excluded to control for confounding factors. It should still be noted that patients undergoing non-NHS treatment, for example knee arthroplasty in the private sector after a previous knee arthroscopy under NHS care, would not be captured in this dataset. For all observational studies utilising large datasets there may be some concerns raised about coding accuracy. The data in our study was cleaned prior to analysis, excluding patients where procedures were missing the side of intervention, and cases where date coding errors were identified. Although some other data coding errors are inevitable, data errors in procedure coding would result in hospitals not receiving payment for surgery performed, and this provides a strong incentive for data accuracy with regards to the coding data analysed in this study.

For peer review only

We were able to stratify risk of arthroplasty by a large number of patient factors, but certain procedure specific data is not recorded. Operative factors, such as the affected compartment of the knee and extent of initial cartilage damage before intervention, are not recorded in this database. These factors may be important in determining outcome, for example, there are likely to be differences in long-term outcomes between chondroplasty performed to the tibiofemoral joint in comparison to the patellofemoral joint.[30] Other unmeasured sources of potential confounding include BMI, leg alignment, and radiographic status at the time of intervention. These are important considerations when considering if a patient is suitable for chondral surgery intervention, but the specific impact of these factors on long-term outcomes in this population remains uncertain. Subjective, patient-reported, symptomatic outcome data is not yet available for this cohort and radiographic outcomes are not recorded in the HES database. Instead, our study focussed on the objective, measurable outcome of knee arthroplasty, matched to the same knee as the previous chondroplasty surgery intervention. Although knee arthroplasty represents the end-stage of symptomatic failure for patients with osteoarthritis, it is likely to considerably underestimate the overall health and symptom burden in this cohort. Patients, particularly younger patients, may not have been willing or suitable candidates for knee arthroplasty, and the threshold for arthroplasty may have been much higher for younger age groups or older patients with multiple comorbidities.

The outcome had patients in our study not undergone chondroplasty remains unknown and an answer to this question requires a randomised controlled trial with a non-operative treatment arm. It is important to note that, in general, chondroplasty is a non-specific term that encompasses several techniques for the debridement of cartilage defects.[7] The findings in this paper cannot be generalised to other types of arthroscopic and joint preservation surgery, cartilage repair and regeneration techniques, such as microfracture and autologous chondrocyte implantation.[6,30]

Our study reports the long-term outcomes following chondroplasty in a high-risk cohort of patients with cartilage damage for the first time. Our findings our stratified by a range of patient specific factors and will be important for informed consent and shared decision making between patients and clinicians. Further work is required to optimise treatment selection, however, and additional patient information may allow more accurate prediction of outcome and guide clinical management.

<u>Conclusion</u>

The risk of knee arthroplasty is 17 times greater in patients with a history of knee chondroplasty and in a proportion of patients, there is insufficient benefit to prevent the need for knee arthroplasty within one or two years. These important new data help inform patients and clinicians of the long-term outcomes following this procedure, at the population level, for the first time. Enhanced clinical guidance on the appropriate indications for chondroplasty are required and there is a need for high-quality randomised

For peer review only

studies to determine the relative clinical and cost effectiveness of this intervention in comparison to alternative, including non-surgical, treatments.

For peer review only

Details of contributors

SGFA: concept, methodology, analysis, writing and editing paper, guarantor.

AJRP: writing and editing paper.

AJ: methodology, analysis, editing paper.

DJB: concept, editing paper.

AJP: concept, methodology, editing paper.

Transparency declaration

The lead author (SA) affirms that the manuscript is an honest, accurate, and transparent account of the study being reported; that no important aspects of the study have been omitted; and that any discrepancies from the study as planned (and registered) have been explained.

Competing interests

Andrew Judge has received consultancy fees from Freshfields Bruckhaus Deringer (on behalf of Smith & Nephew Orthopaedics Limited), and is a member of the Data Safety and Monitoring Board (which involved receipt of fees) from Anthera Pharmaceuticals, Inc. All other authors declare no financial relationships with any organisations that might have an interest in the submitted work in the previous three years, no other relationships or activities that could appear to have influenced the submitted work.

Funding

This report is independent research supported by the National Institute for Health Research (NIHR Doctoral Research Fellowship, Mr Simon Abram, DRF-2017-10-030) and NIHR Oxford Biomedical Research Centre (BRC). Andrew Judge is supported by the NIHR Biomedical Research Centre at the University Hospitals Bristol NHS Foundation Trust and the University of Bristol. The views expressed in this publication are those of the authors and not necessarily those of the NHS, the National Institute for Health Research or the Department of Health and Social Care.

Exclusive license

The Corresponding Author has the right to grant on behalf of all authors and does grant on behalf of all authors, a worldwide licence to the Publishers and its licensees in perpetuity, in all forms, formats and media (whether known now or created in the future), to i) publish, reproduce, distribute, display and store the Contribution, ii) translate the Contribution into other languages, create adaptations, reprints, include within collections and create summaries, extracts and/or, abstracts of the Contribution and convert or allow conversion into any format including without limitation audio, iii) create any other derivative work(s) based in whole or part on the on the Contribution, iv) to exploit all subsidiary rights to exploit all subsidiary rights that currently exist or as may exist in the future in the Contribution, v) the inclusion of electronic links from the Contribution to third party material where-ever it may be located; and, vi) licence any third party to do any or all of the above.

Ethical approval

Not required.

For peer review only

REFERENCES

1 Järvinen TLN, Guyatt GH. Arthroscopic surgery for knee pain. *Bmj* 2016;**3934**:i3934. doi:10.1136/bmj.i3934

2 Kirkley A, Birmingham TB, Litchfield RB, *et al.* A Randomized Trial of Arthroscopic Surgery for Osteoarthritis of the Knee. *N Engl J Med* 2008;**359**:1097–107. doi:10.1056/NEJMoa0708333

3 Moseley JB, O'Malley K, Petersen NJ, *et al.* A controlled trial of arthroscopic surgery for osteoarthritis of the knee. *N Engl J Med* 2002;**347**:81–8. doi:10.1056/NEJMoa013259

4 Aaron RK, Skolnick AH, Reinert SE, *et al.* Arthroscopic Débridement for Osteoarthritis of the Knee. *J Bone Jt Surg* 2006;**88**:936–43. doi:10.2106/JBJS.D.02671

5 Palmer J. S, Monk AP, Hopewell S, *et al.* Surgical interventions for early structural knee osteoarthritis. *Cochrane Database Syst Rev* Published Online First: 23 March 2016. doi:10.1002/14651858.CD012128

6 Mistry H, Connock M, Pink J, *et al.* Autologous chondrocyte implantation in the knee: Systematic review and economic evaluation. *Health Technol Assess (Rockv)* 2017;**21**:V-160. doi:10.3310/hta21060

7 Barber FA, Iwasko NG. Treatment of Grade III Femoral Chondral Lesions: Mechanical Chondroplasty Versus Monopolar Radiofrequency Probe. *Arthrosc - J Arthrosc Relat Surg* 2006;**22**:1312–7. doi:10.1016/j.arthro.2006.06.008

8 Allen RT, Tasto JP, Cummings J, *et al.* Meniscal Debridement With an Arthroscopic Radiofrequency Wand Versus an Arthroscopic Shaver: Comparative Effects on Menisci and Underlying Articular Cartilage. *Arthrosc - J Arthrosc Relat Surg* 2006;**22**:385–93. doi:10.1016/j.arthro.2005.12.007

9 Spahn G, Kahl E, Mückley T, *et al.* Arthroscopic knee chondroplasty using a bipolar radiofrequency-based device compared to mechanical shaver: Results of a prospective, randomized, controlled study. *Knee Surgery, Sport Traumatol Arthrosc* 2008;**16**:565–73. doi:10.1007/s00167-008-0506-1

10 Lu Y, Edwards RB, Cole BJ, *et al.* Thermal chondroplasty with radiofrequency energy. An in vitro comparison of bipolar and monopolar radiofrequency devices. *Am J Sports Med* 2001;**29**:42–9.http://www.ncbi.nlm.nih.gov/pubmed/11206255

11 Dandy DJ. Abrasion Chondroplasty. *Arthrosc J Qf Artluvscopic Relat Surg*;**2**:51–3.https://ac.els-cdn.com/S0749806386800111/1-s2.0-S0749806386800111-main.pdf?_tid=b327693c-0123-4191-9c90-921c887f85db&acdnat=1522234548_babad5ec459e1423df55025bdea79d16 (accessed 28 Mar 2018).

12 National Institute for Health and Care Excellence (NICE). Arthroscopic radiofrequency chondroplasty for discrete chondral defects of the knee | Guidance and guidelines | NICE. NICE 2014. https://www.nice.org.uk/guidance/ipg493 (accessed 10 Jan 2018).

13 NHS Digital. Hospital Episode Statistics. http://content.digital.nhs.uk/hes (accessed 4 Dec 2017).

For peer review only

14 NHS Digital. *National clinical coding standards: OPCS-4 (2017)*. Stationery Office 2017.

15 NHS Digital. Hospital Episode Statistics (HES) Analysis Guide. Published Online First: 2015.http://content.digital.nhs.uk/media/1592/HES-analysis-guide/pdf/HES_Analysis_Guide_March_2015.pdf (accessed 4 Dec 2017).

16 HSCIC. Summary Hospital-level Mortality Indicator (SHMI). Indicator Specification. Version 1.25. 2017. https://www.digital.nhs.uk/SHMI

17 Charlson ME, Pompei P, Ales KL, *et al.* A new method of classifying prognostic in longitudinal studies: development and validation. J. Chronic Dis. 1987;**40**:373–83. doi:0021-9681/87

18 Zhang JX, Iwashyna TJ, Christakis NA. The performance of different lookback periods and sources of information for Charlson comorbidity adjustment in Medicare claims. *Med Care* 1999;**37**:1128–39.http://www.ncbi.nlm.nih.gov/pubmed/10549615

19 Noble M, Wright} G, Smith G, *et al.* Measuring multiple deprivation at the small-area level. *Environ Plan A* 2006;**38**:169–85. doi:10.1068/a37168

20 Abram SGF, Judge A, Beard DJ, *et al.* Temporal trends and regional variation in the rate of arthroscopic knee surgery in England: analysis of over 1.7 million procedures between 1997 and 2017. Has practice changed in response to new evidence? *Br J Sports Med* 2018;:bjsports-2018-099414. doi:10.1136/bjsports-2018-099414

21 Loeser RF. Age-Related Changes in the Musculoskeletal System and the Development of Osteoarthritis. *Clin Geriatr Med* 2010;**26**:371–86. doi:10.1016/j.cger.2010.03.002

22 National Institute for Health and Care Excellence. Arthroscopic knee washout, with or without debridement, for the treatment of osteoarthritis (IPG230). NICE 2007. https://www.nice.org.uk/guidance/ipg230 (accessed 8 Jan 2018).

23 Boyd JA, Gradisar IM. Total Knee Arthroplasty After Knee Arthroscopy in Patients Older Than 50 Years. *Orthopedics* 2016;**39**:1–4. doi:10.3928/01477447-20160719-01

24 Judge A, Welton NJ, Sandhu J, *et al.* Equity in access to total joint replacement of the hip and knee in England: cross sectional study. *Bmj* 2010;**341**:c4092–c4092. doi:10.1136/bmj.c4092

25 Adamson J, Ben-Shlomo Y, Chaturvedi N, *et al.* Ethnicity, socio-economic position and gender - Do they affect reported health-care seeking behaviour? *Soc Sci Med* 2003;**57**:895–904. doi:10.1016/S0277-9536(02)00458-6

26 Chaturvedi N, Rai H, Ben-Shlomo Y. Lay diagnosis and health-care-seeking behaviour for chest pain in south Asians and Europeans. *Lancet* 1997;**350**:1578–83. doi:10.1016/S0140-6736(97)06243-0

27 Jiang L, Tian W, Wang Y, *et al.* Body mass index and susceptibility to knee osteoarthritis: A systematic review and meta-analysis. *Jt Bone Spine* 2012;**79**:291–7. doi:10.1016/j.jbspin.2011.05.015

28 Khan T, Alvand A, Prieto-Alhambra D, *et al.* ACL and meniscal injuries increase the risk of primary total knee replacement for osteoarthritis: a matched case–control study using the Clinical Practice Research Datalink (CPRD). *Br J Sports Med* 2018;:bjsports-2017-097762. doi:10.1136/bjsports-2017-097762

For peer review only

29 Chan SW, Tulloch E, Cooper ES, *et al.* Montgomery and informed consent: where are we now? *BMJ* 2017;**2224**:j2224. doi:10.1136/bmj.j2224

30 Kraeutler MJ, Belk JW, Purcell JM, *et al.* Microfracture Versus Autologous Chondrocyte Implantation for Articular Cartilage Lesions in the Knee: A Systematic Review of 5-Year Outcomes. *Am J Sports Med* 2017;:036354651770191. doi:10.1177/0363546517701912

For peer review only

TABLES

TABLE 1: Demographics and descriptive statistics of chondroplasty cohort

	n	%
**Total**	157730	100.00
**Sex**		
Male	81884	51.91
Female	75846	48.09
**Age group (years)**		
<20	2868	1.82
20-39	24648	15.63
40-59	83258	52.79
60-79	45191	28.65
80+	1765	1.12
**Charlson comorbidity index**		
0	121605	77.10
1 - 15	34719	22.01
16 - 30	1296	0.82
31 - 50	110	0.07
**Index of multiple deprivation (quintiles)**		
1 = least deprived	36043	23.21
2	35189	22.66
3	32493	20.92
4	27312	17.59
5 = most deprived	24266	15.62
Missing	2427	
**Rurality**		
Urban	119766	76.42
Rural	36953	23.58
Missing	1011	
**Ethnicity**		
White	141928	94.43
Asian	953	0.63
Black	4511	3.00
Mixed	2122	1.41
Other	792	0.53
Missing	7424	
**Concurrent procedures**		
None	65987	41.84
Meniscal	91743	58.16

For peer review only

Table 2: Cohort demographics and adjusted odds of arthroplasty

	1-year outcome			2-year outcome			5-year outcome			8-year outcome		
	n	n TKA	% (95% CI)	n	n TKA	% (95% CI)	n	n TKA	% (95% CI)	n	n TKA	% (95% CI)
**Total**	135,197	7984	5.91% (5.78, 6.03)	114,592	10787	9.41% (9.24, 9.58)	57,267	8145	14.22% (13.94, 14.51)	16,347	2879	17.61% (17.03, 18.20)
**Sex**												
Male	69,787	3160	4.53% (4.37, 4.68)	59,101	4261	7.21% (7.00, 7.42)	29,688	3315	11.17% (10.81, 11.53)	8,514	1208	14.19% (13.45, 14.95)
Female	65,410	4824	7.38% (7.18, 7.58)	55,491	6526	11.76% (11.49, 12.03)	27,579	4830	17.51% (17.07, 17.97)	7,833	1671	21.33% (20.43, 22.26)
**Sex**												
< 20	-	-	-	-	-	-	-	-	-	-	-	-
20 - 39	21,548	48	0.22% (0.16, 0.30)	18,583	79	0.43% (0.34, 0.53)	10,004	95	0.95% (0.77, 1.16)	3,094	53	1.71% (1.29, 2.23)
40 - 59	72,345	2654	3.67% (3.53, 3.81)	60,974	4049	6.64% (6.44, 6.84)	29,844	3327	11.15% (10.79, 11.51)	8,552	1287	15.05% (14.30, 15.82)
60 - 79	39,741	4994	12.57% (12.24, 12.90)	33,680	6343	18.83% (18.42, 19.25)	16,716	4522	27.05% (26.38, 27.73)	4,508	1479	32.81% (31.44, 34.20)
80 +	1,563	288	18.43% (16.53, 20.44)	1,355	316	23.32% (21.09, 25.67)	703	201	28.59% (25.28, 32.09)	193	60	31.09% (24.64, 38.13)
**Charlson comorbidity index**												
0	104,530	5369	5.14% (5.00, 5.27)	89,081	7366	8.27% (8.09, 8.45)	45,505	5837	12.83% (12.52, 13.14)	13,362	2148	16.08% (15.46, 16.71)
1 - 15	29,475	2467	8.37% (8.06, 8.69)	24,540	3228	13.15% (12.73, 13.58)	11,371	2207	19.41% (18.69, 20.15)	2,884	701	24.31% (22.75, 25.91)
16 - 30	1,102	138	12.52% (10.63, 14.62)	898	180	20.04% (17.47, 22.82)	391	101	25.83% (21.56, 30.47)	101	30	29.70% (21.02, 39.61)
31 - 50	90	10	11.11% (5.46, 19.49)	73	13	17.81% (9.84, 28.53)	-	-	-	-	-	-
**Index of multiple deprivation (quintiles)**												
1	31,054	1846	5.94% (5.68, 6.21)	26,546	2405	9.06% (8.72, 9.41)	13,422	1835	13.67% (13.09, 14.26)	3,878	629	16.22% (15.07, 17.42)
2	30,218	1799	5.95% (5.69, 6.23)	25,638	2409	9.40% (9.04, 9.76)	12,819	1779	13.88% (13.28, 14.49)	3,474	617	17.76% (16.50, 19.07)
3	27,974	1737	6.21% (5.93, 6.50)	23,721	2324	9.80% (9.42, 10.18)	11,833	1772	14.98% (14.34, 15.63)	3,374	635	18.82% (17.51, 20.18)
4	23,312	1387	5.95% (5.65, 6.26)	19,702	1913	9.71% (9.30, 10.13)	9,771	1420	14.53% (13.84, 15.25)	2,762	496	17.96% (16.54, 19.44)
5	20,591	1104	5.36% (5.06, 5.68)	17,194	1588	9.24% (8.81, 9.68)	8,420	1197	14.22% (13.48, 14.98)	2,451	444	18.12% (16.61, 19.70)
**Rurality**												
Urban	102,665	6004	5.85% (5.71, 5.99)	86,807	8135	9.37% (9.18, 9.57)	43,287	6148	14.20% (13.88, 14.54)	2,154	12,242	17.60% (16.92, 18.28)
Rural	31,760	1944	6.12% (5.86, 6.39)	27,127	2613	9.63% (9.28, 9.99)	13,739	1980	14.41% (13.83, 15.01)	713	3,987	17.88% (16.70, 19.11)
**Ethnicity**												
White	122,261	7672	6.28% (6.14, 6.41)	103,979	10366	9.97% (9.79, 10.15)	52,267	7834	14.99% (14.68, 15.30)	14,908	2,750	18.45% (17.83, 19.08)
Mixed	750	21	2.80% (1.74, 4.25)	609	32	5.25% (3.62, 7.34)	278	22	7.91% (5.03, 11.74)	76	8	10.53% (4.66, 19.69)
Asian	3,722	130	3.49% (2.93, 4.13)	3,088	186	6.02% (5.21, 6.92)	1,465	167	11.40% (9.82, 13.14)	362	75	20.72% (16.66, 25.26)
Black	1,770	27	1.53% (1.01, 2.21)	1,466	53	3.62% (2.72, 4.70)	677	38	5.61% (4.00, 7.62)	171	15	8.77% (4.99, 14.06)
Other	645	15	2.33% (1.31, 3.81)	518	18	3.47% (2.07, 5.44)	250	21	8.40% (5.27, 12.55)	75	9	12.00% (5.64, 21.56)
**Concurrent procedures**												
None	57,208	2686	4.70% (4.52, 4.87)	50,256	3754	7.47% (7.24, 7.70)	28,578	3252	11.38% (11.01, 11.75)	9,370	1,389	14.82% (14.11, 15.56)
Meniscal	77,989	5298	6.79% (6.62, 6.97)	64,336	7033	10.93% (10.69, 11.18)	28,689	4893	17.06% (16.62, 17.50)	6,977	1,490	21.36% (20.40, 22.34)

- = suppressed due to small numbers; CI = confidence interval; TKA = total or partial knee arthroplasty

For peer review only

Table 3: Unadjusted and adjusted* risk of knee arthroplasty following arthroscopic chondroplasty

	Unadjusted Risk Subsequent TKA		Adjusted Risk Subsequent TKA	
	**HR**	**95% CI**	**HR**	**95% CI**
**Sex**				
Male	1.00	1.00	1.00	1.00
Female	1.61	1.57, 1.66	1.38	1.34, 1.42
**Age (per five years) ‡**				
Per year	1.35	1.35, 1.36	1.33	1.32, 1.34
**Year of treatment (per five years)**				
Year	0.99	0.96, 1.03	0.95	0.92, 0.98
**Charlson comorbidity index (per five units)**				
Charlson index	1.29	1.27, 1.31	1.03	1.01, 1.05
**Index of multiple deprivation (quintile)**				
1 = least	1.00	1.00	1.00	1.00
2	1.03	0.99, 1.08	1.07	1.03, 1.12
3	1.08	1.04, 1.13	1.17	1.12, 1.22
4	1.03	0.99, 1.08	1.20	1.15, 1.26
5 = most	1.01	0.96, 1.06	1.29	1.23, 1.36
**Rurality**				
Urban	1.00	1.00	1.00	1.00
Rural	1.03	1.00, 1.07	0.99	0.95, 1.02
**Ethnicity**				
White	1.00	1.00	1.00	1.00
Mixed	0.50	0.38, 0.65	0.66	0.51, 0.86
Asian	0.65	0.59, 0.72	0.73	0.66, 0.81
Black	0.35	0.28, 0.42	0.44	0.36, 0.54
Other	0.34	0.24, 0.48	0.45	0.32, 0.64
**Concurrent procedures**				
None	1.00	1.00	1.00	1.00
Meniscal surgery	1.52	1.48, 1.57	1.09	1.06, 1.13

** adjusted by all variables in the table; ‡ age <20 years suppressed due to small numbers; HR = hazard ratio; CI = confidence interval; TKA = total or partial knee arthroplasty*

For peer review only

Table 4: Rates and relative risk of undergoing TKA with previous chondroplasty by age at TKA in 2016-17

	**Prior chondroplasty**		**Without prior chondroplasty**		**Relative Risk**	
**Age at TKA (years)**	**Annual Rate TKA /100k**	**95% CI**	**Annual Rate TKA /100k**	**95% CI**	**RR**	**95% CI**
30 - 39	274.48 (0.27%)	190.16, 383.35 (0.19%, 0.38%)	1.60 (0.00%)	1.32, 1.92 (0.00%, 0.00%)	170.92	116.72, 250.30
40 - 49	1454.02 (1.45%)	1318.04, 1600.06 (1.32%, 1.60%)	19.79 (0.02%)	18.79, 20.82 (0.02%, 0.02%)	72.45	65.00, 80.76
50 - 59	3626.62 (3.63%)	3448.20, 3811.60 (3.45%, 3.81%)	130.68 (0.13%)	128.05, 133.35 (0.13%, 0.13%)	26.82	25.41, 28.30
60 - 69	5179.17 (5.18%)	4933.67, 5433.20 (4.93%, 5.43%)	386.68 (0.39%)	381.64, 391.77 (0.38%, 0.39%)	12.78	12.16, 13.44
70 +	6090.50 (6.09%)	5721.53, 6475.82 (5.72%, 6.48%)	520.46 (0.52%)	514.89, 526.07 (0.51%, 0.53%)	11.09	10.42, 11.80
Overall (30 +)	3494.61 (3.49%)	3394.82, 3596.52 (3.39%, 3.60%)	195.38 (0.20%)	193.90, 196.87 (0.19%, 0.20%)	17.32	16.81, 17.84

TKA = total or partial knee arthroplasty; RR = risk ratio

For peer review only

FIGURES

Figure 1: Flow chart illustrating extraction of patient level cohort

For peer review only

Figure 2: Survival curve (not undergoing knee arthroplasty) following chondroplasty by age†

† Age groups < 20 years and 80+ years suppressed due to small numbers

For peer review only

Figure 3: Survival curve (not undergoing knee arthroplasty) following chondroplasty by sex*

For peer review only

** Age groups < 20 years and 80+ years suppressed due to small numbers*

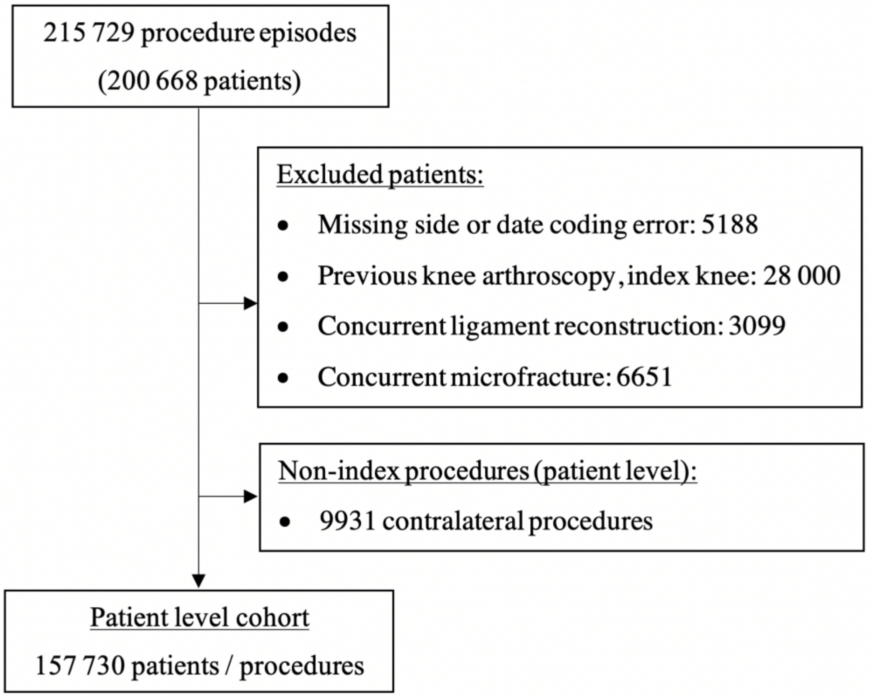

Flow chart illustrating extraction of patient level cohort

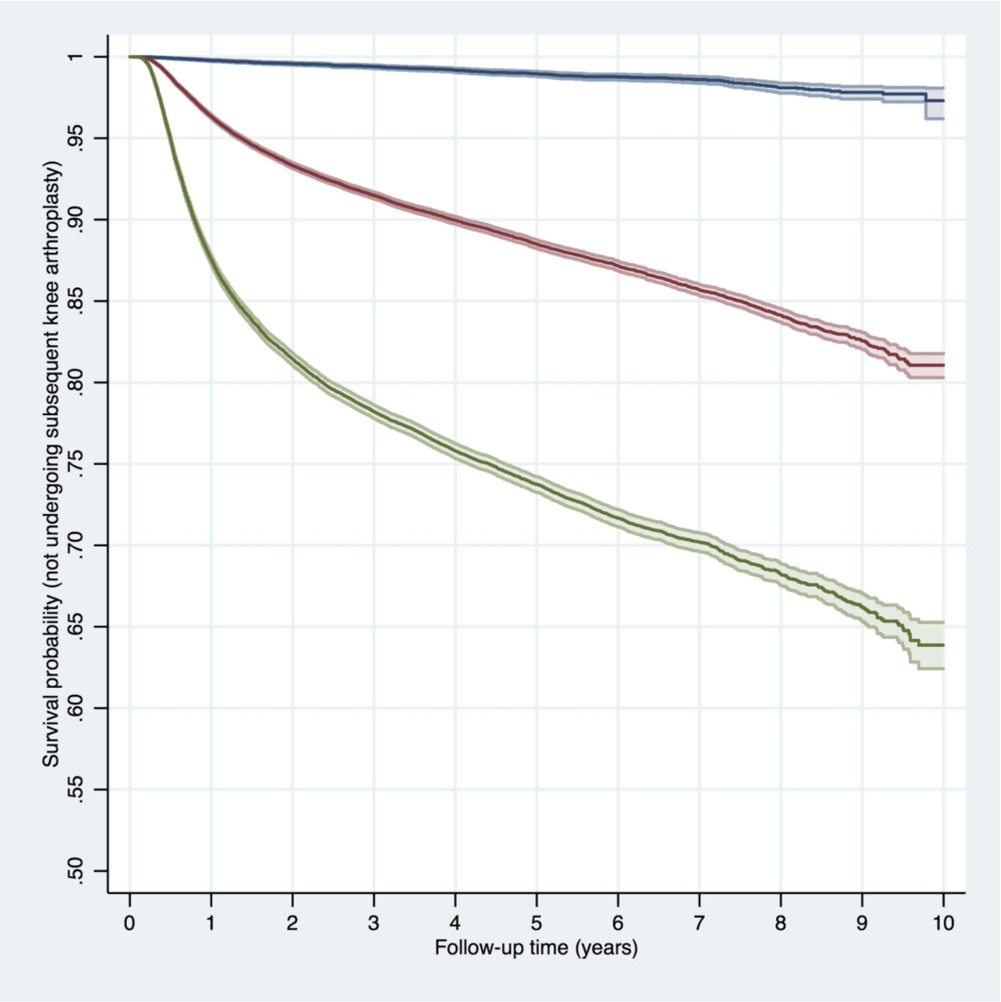

Survival curve (not undergoing knee arthroplasty) following chondroplasty by age

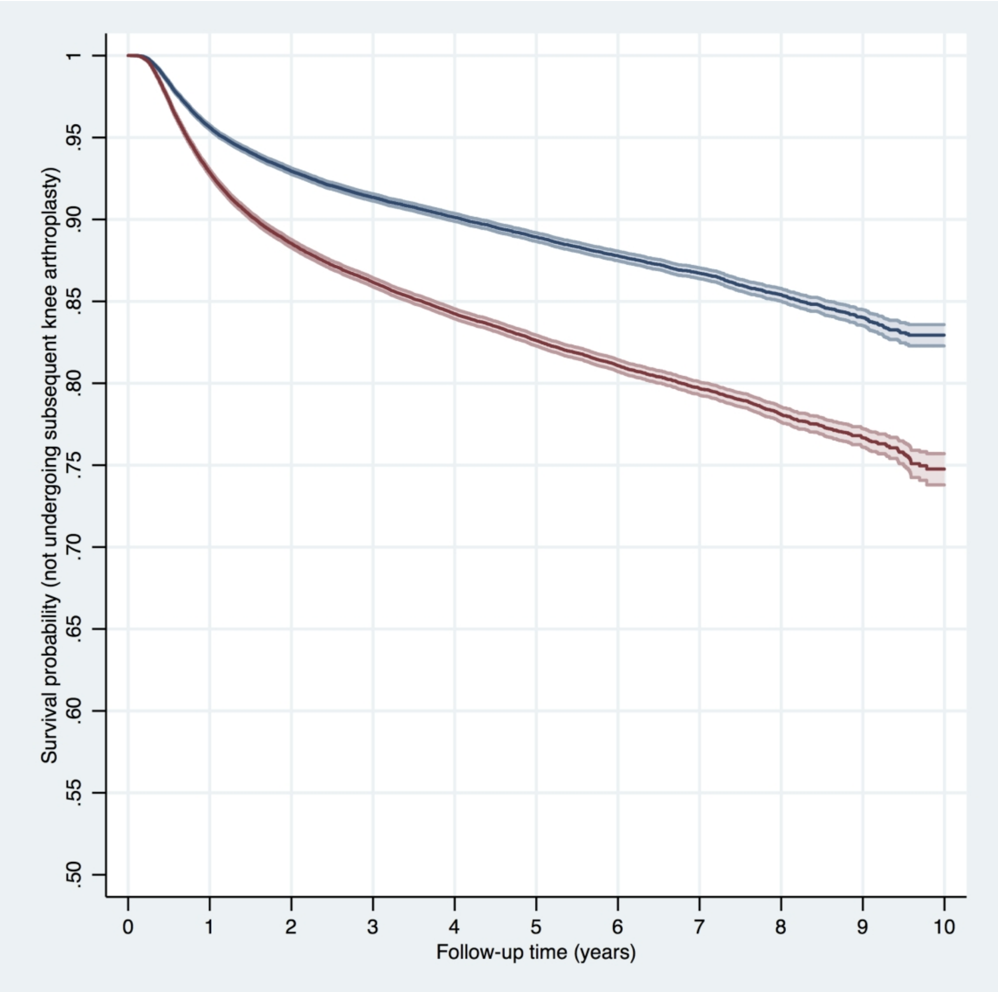

Survival curve (not undergoing knee arthroplasty) following chondroplasty by sex

Appendix 1: OPCS procedure code list

**PROCEDURE**	**OPCS 4.4**	**OPCS 4.5**	**OPCS 4.6**	**OPCS 4.7**	**Description**
Chondroplasty*	W83.3*	W83.3*	W83.3*	W83.3*	Endoscopic shaving of articular cartilage
Chondroplasty*	W83.4*	W83.4*	W83.4*	W83.4*	Endoscopic articular abrasion chondroplasty
Chondroplasty*	W83.5*	W83.5*	W83.5*	W83.5*	Endoscopic articular thermal chondroplasty
Chondroplasty*	W83.6*	W83.6*	W83.6*	W83.6*	Endoscopic excision of articular cartilage NEC
Chondroplasty*	W89.1*	W89.1*	W89.1*	W89.1*	Endoscopic chondroplasty NEC
Microfracture*	W83.1*	W83.1*	W83.1*	W83.1*	Endoscopic drilling of lesion of articular cartilage
Microfracture*	W84.5*	W84.5*	W84.5*	W84.5*	Endoscopic drilling of epiphysis for repair of articular cartilage
Meniscal surgery	W82.2	W82.2	W82.2	W82.2	Endoscopic resection of semilunar cartilage NEC
Meniscal surgery	W82.3	W82.3	W82.3	W82.3	Endoscopic repair of semilunar cartilage
Meniscal surgery	W82.1	W82.1	W82.1	W82.1	Endoscopic total excision of semilunar cartilage
Ligament reconstruction (exclusion)*	W74.2	W74.2	W74.2	W74.2	Reconstruction of intra-articular ligament NEC
Ligament reconstruction (exclusion)*	W84.1	W84.1	W84.1	W84.1	Endoscopic repair of intra-articular ligament
Ligament reconstruction (exclusion)*	W84.2	W84.2	W84.2	W84.2	Endoscopic reattachment of intra-articular ligament
Ligament reconstruction (exclusion)*	W72.3	W72.3	W72.3	W72.3	Primary prosthetic replacement of intra-articular ligament
Ligament reconstruction (exclusion)*	W72.4	W72.4	W72.4	W72.4	Prosthetic replacement of intra-articular ligament NEC
Arthroplasty*	W43.1	W43.1	W43.1	W43.1	Primary total prosthetic replacement of joint using cement NEC
Arthroplasty*	W44.1	W44.1	W44.1	W44.1	Primary total prosthetic replacement of joint not using cement NEC
Arthroplasty*	W44.8	W44.8	W44.8	W44.8	Other specified total prosthetic replacement of other joint not using cement
Arthroplasty*	W44.9	W44.9	W44.9	W44.9	Unspecified total prosthetic replacement of other joint not using cement
Arthroplasty*	W45.1	W45.1	W45.1	W45.1	Primary total prosthetic replacement of joint NEC
Arthroplasty*	W45.8	W45.8	W45.8	W45.8	Other specified other total prosthetic replacement of other joint
Arthroplasty*	W45.9	W45.9	W45.9	W45.9	Unspecified other total prosthetic replacement of other joint
Arthroplasty*	W53.8	W53.8	W53.8	W53.8	Other specified prosthetic replacement of articulation of other bone not using cement
Arthroplasty	W40.1	O18.1	O18.1	O18.1	Primary hybrid prosthetic replacement of knee joint using cement
Arthroplasty	W40.8	O18.8	O18.8	O18.8	Other specified hybrid prosthetic replacement of knee joint using cement
Arthroplasty	W40.9	O18.9	O18.9	O18.9	Unspecified hybrid prosthetic replacement of knee joint using cement
Arthroplasty	W40.1	W40.1	W40.1	W40.1	Primary total prosthetic replacement of knee joint using cement
Arthroplasty	W40.8	W40.8	W40.8	W40.8	Other specified total prosthetic replacement of knee joint using cement

For peer review only

Arthroplasty	W40.9	W40.9	W40.9	W40.9	Unspecified total prosthetic replacement of knee joint using cement
Arthroplasty	W41.1	W41.1	W41.1	W41.1	Primary total prosthetic replacement of knee joint not using cement
Arthroplasty	W41.8	W41.8	W41.8	W41.8	Other specified total prosthetic replacement of knee joint not using cement
Arthroplasty	W41.9	W41.9	W41.9	W41.9	Unspecified total prosthetic replacement of knee joint not using cement
Arthroplasty	W42.1	W42.1	W42.1	W42.1	Primary total prosthetic replacement of knee joint NEC
Arthroplasty	W42.8	W42.8	W42.8	W42.8	Other specified other total prosthetic replacement of knee joint
Arthroplasty	W42.9	W42.9	W42.9	W42.9	Unspecified other total prosthetic replacement of knee joint
Arthroplasty*	W58.1	W58.1	W58.1	W58.1	Primary resurfacing arthroplasty of joint

**: Additional site-specific knee code required (Z846, Z765, Z845, Z844, Z774, or Z787)*

For peer review only

Appendix 2: Flow chart illustrating the extraction of the knee arthroplasty cohort

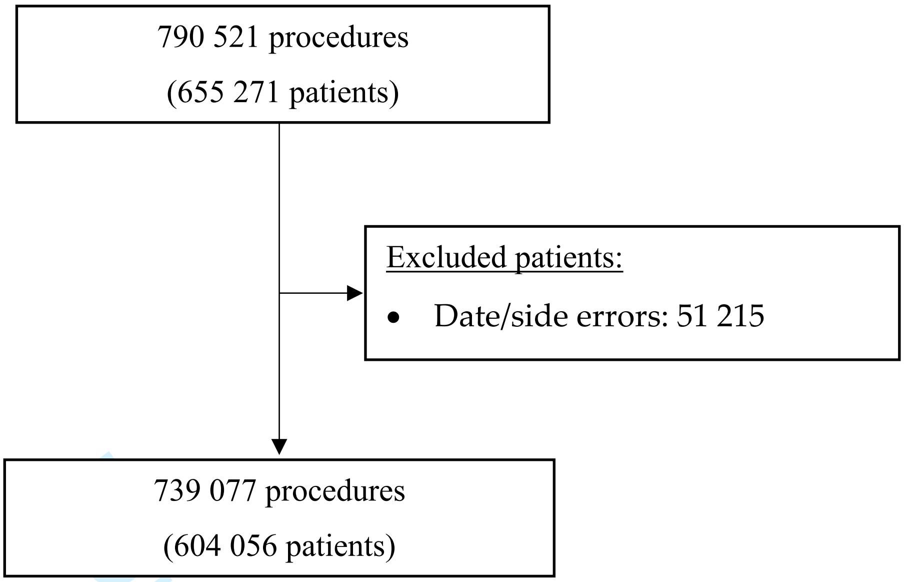

For peer review only

Rates of knee arthroplasty in patients with a history of arthroscopic chondroplasty: results from a retrospective cohort study utilising the National Hospital Episode Statistics for England

Journal:	*BMJ Open*
Manuscript ID	bmjopen-2019-030609.R1
Article Type:	Original research
Date Submitted by the Author:	23-Oct-2019

Complete List of Authors:	Abram, Simon; University of Oxford Nuffield Department of Orthopaedics Rheumatology and Musculoskeletal Sciences,<br>Palmer, Antony; University of Oxford, Nuffield Department of Orthopaedics, Rheumatology and Musculoskeletal Sciences<br>Judge, Andrew; University of Bristol, Musculoskeletal Research Unit, Translational Health Sciences, Bristol Medical School, University of Bristol, Learning and Research Building, Level 1, Southmead Hospital, Southmead, BS10 5NB<br>Beard, David; University of Oxford, Nuffield Dept of Orthopaedics, Rheumatology and Musculoskeletal Sciences<br>Price, Andrew; University of Oxford, Nuffield Department of Orthopaedics, Rheumatology and Musculoskeletal Sciences

**Primary Subject Heading**:	Surgery
Secondary Subject Heading:	Rheumatology, Sports and exercise medicine, Epidemiology
Keywords:	Knee < ORTHOPAEDIC & TRAUMA SURGERY, arthroscopy, chondroplasty, knee replacement, arthroplasty, osteoarthritis

I, the Submitting Author has the right to grant and does grant on behalf of all authors of the Work (as defined in the below author licence), an exclusive licence and/or a non-exclusive licence for contributions from authors who are: i) UK Crown employees; ii) where BMJ has agreed a CC-BY licence shall apply, and/or iii) in accordance with the terms applicable for US Federal Government officers or employees acting as part of their official duties; on a worldwide, perpetual, irrevocable, royalty-free basis to BMJ Publishing Group Ltd ("BMJ") its licensees and where the relevant Journal is co-owned by BMJ to the co-owners of the Journal, to publish the Work in this journal and any other BMJ products and to exploit all rights, as set out in our licence.

The Submitting Author accepts and understands that any supply made under these terms is made by BMJ to the Submitting Author unless you are acting as an employee on behalf of your employer or a postgraduate student of an affiliated institution which is paying any applicable article publishing charge ("APC") for Open Access articles. Where the Submitting Author wishes to make the Work available on an Open Access basis (and intends to pay the relevant APC), the terms of reuse of such Open Access shall be governed by a Creative Commons licence – details of these licences and which Creative Commons licence will apply to this Work are set out in our licence referred to above.

Other than as permitted in any relevant BMJ Author's Self Archiving Policies, I confirm this Work has not been accepted for publication elsewhere, is not being considered for publication elsewhere and does not duplicate material already published. I confirm all authors consent to publication of this Work and authorise the granting of this licence.

For peer review only

Rates of knee arthroplasty in patients with a history of arthroscopic chondroplasty: results from a retrospective cohort study utilising the National Hospital Episode Statistics for England

1. S.G.F. Abram, MRCS[1,2]
2. A.J.R. Palmer, DPhil[1,2]
3. A. Judge, PhD[1,3,4]
4. D.J. Beard, DPhil[1,2]
5. A.J. Price, DPhil[1,2]

Author affiliations:

[1]Nuffield Department of Orthopaedics, Rheumatology and Musculoskeletal Sciences, University of Oxford
[2]NIHR Biomedical Research Centre, Oxford
[3]Musculoskeletal Research Unit, University of Bristol
[4]NIHR Biomedical Research Centre, Bristol

Author titles:

SGFA: NIHR Doctoral Research Fellow
AJRP: NIHR Clinical Lecturer
AJ: Professor of Translational Statistics
DJB: Professor of Musculoskeletal Sciences
AJP: Professor of Orthopaedics

Corresponding author:

Simon Abram

E: simon.abram@ndorms.ox.ac.uk

T: 01865 223425

Botnar Research Centre, University of Oxford

Nuffield Department of Orthopaedics, Rheumatology and Musculoskeletal Sciences

Windmill Road, Oxford OX3 7LD. UK

Keywords

knee; arthroscopy; chondroplasty; knee replacement; arthroplasty; outcomes; osteoarthritis

Word count

2601

For peer review only

ABSTRACT

OBJECTIVE

The purpose of this study was to analyse the rate of knee arthroplasty in the population of patients with a history of arthroscopic chondroplasty of the knee, in England, over ten years, with comparison to general population data for patients without a history of chondroplasty.

DESIGN

Retrospective cohort study

SETTING

English hospital episode statistics (HES) data

PARTICIPANTS AND INTERVENTIONS

Patients undergoing arthroscopic chondroplasty in England between 2007/08 and 2016/17 were identified. Patients undergoing previous arthroscopic knee surgery or simultaneous cruciate ligament reconstruction or microfracture in the same knee were excluded.

OUTCOMES

Patients subsequently undergoing a knee arthroplasty in the same knee were identified and mortality-adjusted survival analysis was performed (survival without undergoing knee arthroplasty). A Cox proportional hazards model was used to identify factors associated with knee arthroplasty. Relative risk of knee arthroplasty (total or partial) in comparison to the general population was determined.

RESULTS

Through 2007-17, 157,730 eligible chondroplasty patients were identified. Within one-year, 5.91% (7984/135197; 95% CI 5.78 to 6.03) underwent knee arthroplasty and 14.22% (8145/57267; 95% CI 13.94 to 14.51) within five-years. Patients aged over 30 years with a history of chondroplasty were 17.32 times (risk ratio; 95% CI 16.81 to 17.84) more likely to undergo arthroplasty than the general population without a history of chondroplasty.

CONCLUSIONS

Patients with cartilage lesions of the knee, treated with arthroscopic chondroplasty, are at greater risk of subsequent knee arthroplasty than the general population and for a proportion of patients, there is insufficient benefit to prevent the need for knee arthroplasty within one- to five-years. These important new data will inform patients of the anticipated outcomes following this procedure. The risk in comparison to non-operative treatment remains unknown and there is an urgent need for a randomised clinical trial in this population.

For peer review only

STRENGTHS AND LIMITATIONS OF THIS STUDY

- Strengths of the data source analysed in this study include comprehensive, national, data collection and the ability to match treatment with outcomes, including by the laterality of intervention, over time.
- This is the largest cohort of patients undergoing arthroscopic chondroplasty that has been reported, with strict inclusion criteria, excluding patients with a history of previous surgery to the same knee and those undergoing simultaneous ligament reconstruction or microfracture.
- All studies of this design rely on coding accuracy and some coding errors are inevitable; although outcomes were stratified by a range of patient factors, unmeasured potential confounders include body mass index, limb alignment, baseline radiographic status.
- Knee arthroplasty is an end-stage outcome and will underestimate the true burden and severity of symptomatic osteoarthritis in this population.
- The outcome had these patients not undergone arthroscopic chondroplasty remains unknown.

For peer review only

INTRODUCTION

Around 2 million knee arthroscopy procedures are performed worldwide each year.[1] Historically, knee washout and 'debridement' was shown to be ineffective for the treatment of advanced osteoarthritis.[2–4] For early osteoarthritis, however, a number of surgical and non-surgical treatments are available and treatment selection is challenging.[5] The aim of treatment in these cases is to improve symptoms and delay or prevent progressive osteoarthritis.[6]

Chondroplasty is a non-specific term that encompasses several techniques for the treatment of cartilage defects.[7] It includes debridement and abrasion using mechanical 'shavers' and, more recently, thermal or radiofrequency techniques have also emerged despite some concerns these techniques might risk inducing localised chondrocyte death.[7–11] Recent national guidance was cautiously supportive of radiofrequency chondroplasty for the treatment of "discrete chondral defects" based on a small number of clinical trials comparing the outcomes of mechanical and radiofrequency techniques.[12] It is not known which patients are most likely to benefit from chondroplasty procedures and when the procedure does not provide sustained benefit, knee arthroplasty is often indicated. The success rate of chondroplasty is, however, poorly understood and the proportion of patients undergoing subsequent knee arthroplasty after this intervention has been unknown.

The purpose of this study was to determine the proportion of patients undergoing knee chondroplasty procedures that subsequently receive a knee arthroplasty in the same knee, with specific focus on the proportion of patients undergoing early arthroplasty with one-year or two-years of chondroplasty. Factors associated with the risk of subsequent arthroplasty are reported and the relative risk in comparison to the general population determined.

For peer review only

METHODS

Data source

National Hospital Episode Statistics (HES) data was obtained (application DARS-NIC-68703). HES contains a record of all patient attendances at National Health Service (NHS) hospitals in England.[13] The data is submitted by hospitals to claim payment for the services they provide and is also intended for secondary use, including research. HES includes episodes of care delivered in treatment centres (including those in the independent sector) funded by the NHS, episodes of care in England where patients are resident outside of England, and privately funded patients treated within NHS England hospitals. The information recorded in the HES database includes patient demographic and residence data, primary and secondary diagnoses including comorbidities, and all procedures undertaken.

Procedures

All HES records between 1 April 2007 and 31 March 2017 were extracted for patients undergoing arthroscopic chondroplasty. Patients undergoing previous arthroscopic knee surgery or simultaneous cruciate ligament reconstruction or microfracture in the same knee were excluded. Procedures were identified using the Classification of Surgical Operations and Procedures (OPCS-4) codes recorded within the HES data (see appendix 1 for OPCS-4 code list).[14] All knee arthroplasty (partial or total) procedures were also identified (appendix 2) for the whole population to enable the relative risk of knee arthroplasty with and without a history of chondroplasty to be determined.

Outcomes

The primary outcome was knee arthroplasty, matched to the side of any previous chondroplasty (using recorded OPCS-4 laterality codes).

Statistical analysis

Stata v15.1 (StataCorp, College Station, Texas, USA) was used to perform all analysis. In accordance with Office for National Statistics (ONS) and NHS Digital guidance, rates where the number of events was less than six were suppressed.[15] Procedures with date errors or missing laterality were excluded. The absolute rate of knee arthroplasty was determined at 1 year, 2 years, 5 years, and 8 years following arthroscopic chondroplasty as the proportion of the cohort with this minimum period of follow-up. Mortality adjusted Kaplan-Meier survival analysis (survival was defined as not undergoing knee arthroplasty) was also performed and stratified by patient age group and sex.

A Cox proportional-hazards model was used first to calculate the unadjusted hazard ratio of knee arthroplasty over time by age group, sex, index of multiple deprivation (quintile derived from regional factors in England including average income, employment, education, housing, and crime; 1=least deprived

For peer review only

area, 5=most deprived), ethnicity, modified Charlson comorbidity index (derived with maximum 5-year diagnosis code lookback period),[16–18] year of treatment (chondroplasty), rurality, and ethnicity respectively.[16–19] The hazard ratios were then adjusted including all these variables in the model.

The relative risk (risk ratio) of knee arthroplasty in the population of patients with a history of chondroplasty in comparison to the general population (without a history of chondroplasty) was estimated for the year 2016-17. All patients undergoing knee arthroplasty in 2016-17 were identified and the number of these patients with a recorded previous chondroplasty (in the prior 10-years of HES data), versus those without, made up the numerator for each respective population. The chondroplasty population denominator was the number of patients with a history of chondroplasty that had not undergone a knee arthroplasty prior to 2016-17. The denominator for the non-chondroplasty population was the ONS mid-year population estimate less the chondroplasty population.

Patient and public involvement

There was no patient and public involvement in this study.

For peer review only

RESULTS

Over the study period, 157,730 chondroplasty patients were identified as eligible for analysis (Figure 1). The mean age of the chondroplasty cohort was 51.7 year (SD 13.8) and 48.1% were female (Table 1). Over the same period, 604,056 patients underwent knee arthroplasty, of which 35,916 (5.95%) had a record of a previous chondroplasty (Table 1).

Overall, following chondroplasty, 5.91% (7984/135197; 95% confidence interval [CI] 5.78 to 6.03) patients underwent knee arthroplasty within 1 year, 9.41% (10787/114592; 95% CI 9.24 to 9.58) within 2 years, 14.22% (8145/57267; 95% CI 13.94 to 14.51) within 5 years, and 17.61% (2879/16347; 95% CI 17.03 to 18.20) within 8 years (Table 2). The risk of arthroplasty was greater in female patients (adjusted hazard ratio [HR] 1.38; 95% CI 1.34 to 1.42) and in older patients (adjusted HR 1.33 per five years; 95% CI 1.32 to 1.34) (Table 3, Figure 2; Figure 3). Patients with a greater comorbidity index were also at increased risk of subsequently undergoing arthroplasty (adjusted HR 1.03 per five units Charlson index; 95% CI 1.01 to 1.05).

The risk of knee arthroplasty after chondroplasty fell slightly over time, by year of chondroplasty treatment (adjusted HR 0.95 per five years; 95% CI 0.92 to 0.98). Patients in regions of increased deprivation and patients of white ethnicity were at greater risk of subsequent arthroplasty (Table 2). Patients undergoing concurrent meniscal surgery were also at greater risk of subsequent arthroplasty (adjusted HR 1.09; 95% CI 1.06 to 1.13).

In 2016-17, the rate of knee arthroplasty was 3.49% (95% CI 3.39 to 3.60) in patients (aged 30 or older) with a recorded history of chondroplasty and 0.20% (95% CI 0.19 to 0.20) in patients without a record of chondroplasty. This corresponded to an overall relative risk of knee arthroplasty for the chondroplasty cohort patients of 17.32 times (risk ratio [RR]; 95% CI 16.81 to 17.84) that of the general population (Table 4).

Although the absolute annual rate of knee arthroplasty was low, the relative risk of undergoing knee arthroplasty at a younger age was greatly elevated in comparison to arthroplasty at an older age, as shown in Table 4. Patients aged 30-39 with a history of a previous chondroplasty were 170.92 times (RR; 95% CI 116.72 to 250.30) more likely to undergo knee arthroplasty than the general population, per year, in comparison to 11.09 times (RR; 95% CI 10.42 to 11.80) more likely for the over 69 age group.

For peer review only

DISCUSSION

Principal findings

Patients undergoing chondroplasty procedures of the knee have a 17 times increased risk of receiving a knee arthroplasty compared with the general population. Nearly 10% of patients will have received a knee arthroplasty within two years of the chondroplasty procedure. The relative risk of undergoing arthroplasty at a young age is particularly elevated, reaching 171 times the general population rate for arthroplasty between the ages of 30 and 39 years of age. For a proportion of patients, the results indicate insufficient benefit to prevent the need for knee arthroplasty within one or two years, but the risk had these patients not undergone chondroplasty remains unknown.

Comparison to other studies

We previously reported trends in chondroplasty surgery in England, but data from other countries is not available.[20] The age-sex standardised rate of chondroplasty increased 191% from 17.6/100,000 (95% CI 17.2 to 18.0) in 2007/08 to 51.2/100,000 (95% CI 50.6 to 51.7) in 2016/17.[20] The rate of chondroplasty was greatest in patients aged 40-59 years (increasing 210% from 34.3/100,000 in 2007/08 to 106.4/100,000 in 2016/17.[20]

In England, although national guidance has been cautiously supportive of radiofrequency chondroplasty for specific indications, there is only limited evidence demonstrating the effectiveness of chondroplasty compared with alternative surgical or non-surgical treatments.[12] The only randomised studies have been limited to comparisons of different chondroplasty techniques.[10,12] Long-term outcomes following chondroplasty have yet to be reported.[12]

Older patients are much more likely to have generalised osteoarthritis, rather than “discrete chondral defects” for which the national guidance supports radiofrequency chondroplasty.[12,21] For more generalised osteoarthritis, chondroplasty is analogous to debridement and washout, where multiple clinical trials demonstrate no benefit.[2–4] The use of chondroplasty in the treatment of patients with more generalised chondral pathology is therefore unproven and not recommended.[22] In our study, there was considerable age-group variation in outcomes, with 18.8% of patients aged 60-79-years undergoing arthroplasty within two-years of chondroplasty, in comparison to 0.43% for patients undergoing chondroplasty aged 20-39-years. This observation is consistent with the presence of more established osteoarthritis in older age groups.

Our study represents a high-risk cohort of patients with cartilage damage. It is unknown from this observational data whether undergoing the chondroplasty procedure was beneficial to the symptoms or prognosis of these individuals over the full study period. Patients undergoing arthroplasty within 1-year of

For peer review only

their arthroscopic chondroplasty, however, suggests sub-optimal treatment selection. These individuals are highly unlikely to have had only localised or partial thickness lesions and our results may indicate that knee arthroplasty may have been a more appropriate treatment. It is unlikely that chondroplasty in these patients was a cost-effective choice of intervention, but whether chondroplasty can be cost-effective with optimal patient selection is unknown and requires evaluation in a high-quality randomised controlled trial.

Female patients were observed to be of greater risk of subsequent arthroplasty in our study. This has previously been observed following knee arthroscopy in the United States.[23] Patients of white ethnicity and greater deprivation were also at greater risk in our cohort. These findings may reflect differences in healthcare access including treatment thresholds for either the chondroplasty or knee arthroplasty, or differences in care seeking behaviour which has been shown to be influenced by socioeconomic, cultural, occupational, and psychological factors, or there could be biological factors underlying the observation.[24–26] Patients with a greater comorbidity index were more likely to undergo subsequent arthroplasty, and the reason for this is unclear. One possible explanation might be an association between comorbidity and higher body mass index (BMI), which is not recorded in this dataset, with patients having a greater BMI being more likely to progress to end-stage osteoarthritis, or that these patients had more severe pathology at the time of their index chondroplasty.[27] Patients undergoing concurrent meniscal surgery were also more likely to undergo subsequent arthroplasty, which is expected given the association between meniscal injury, osteoarthritis, and knee arthroplasty.[28]

Recently, there has been renewed focus on the importance of and requirements for individualised patient consent.[29] Our findings make an important contribution to the current evidence, and patients can now be appropriately counselled and consented with knowledge of anticipated long-term outcomes.

<u>Strength and limitations</u>

A key strength of our study is the identification of all knee chondroplasty procedures performed in the National Health Service over a 10-year period, creating the largest reported cohort of patients receiving this procedure. Patients with a history of prior arthroscopy in the same knee, simultaneous ligament reconstruction or microfracture were excluded as potential confounding factors. It should still be noted that patients undergoing non-NHS treatment, for example knee arthroplasty in the private sector after a previous knee arthroscopy under NHS care, would not be captured in this dataset and the number of these procedures performed in the private sector is currently unknown. National data does indicate, however, that private healthcare expenditure as a proportion of total healthcare expenditure has remained relatively stable at around 17-18% of total health expenditure between 2005 and 2015.[30] For all observational studies utilising large datasets there may be some concerns raised about coding accuracy. The data in our study was cleaned prior to analysis, excluding patients where procedures were missing the side of intervention, and cases where date coding errors were identified. Although some other data coding errors are inevitable, data

For peer review only

errors in procedure coding would result in hospitals not receiving payment for surgery performed, and this provides a strong incentive for data accuracy with regards to the coding data analysed in this study.

We were able to stratify risk of arthroplasty by a large number of patient factors, but certain procedure specific data is not recorded. Operative factors, such as the affected compartment of the knee and extent of initial cartilage damage before intervention, are not recorded in this database. These factors may be important in determining outcome, for example, there are likely to be differences in long-term outcomes between chondroplasty performed to the tibiofemoral joint in comparison to the patellofemoral joint.[31] Other unmeasured sources of potential confounding include BMI, leg alignment, and radiographic status at the time of intervention. These are important considerations when considering if a patient is suitable for chondral surgery intervention, but the specific impact of these factors on long-term outcomes in this population remains uncertain. Subjective, patient-reported, symptomatic outcome data is not yet available for this cohort and radiographic outcomes are not recorded in the HES database. Instead, our study focussed on the objective, measurable outcome of knee arthroplasty, matched to the same knee as the previous chondroplasty surgery intervention. Although knee arthroplasty represents the end-stage of symptomatic failure for patients with osteoarthritis, it is likely to considerably underestimate the overall health and symptom burden in this cohort. Patients, particularly younger patients, may not have been willing or suitable candidates for knee arthroplasty, and the threshold for arthroplasty may have been much higher for younger age groups or older patients with multiple comorbidities.

The outcome had patients in our study not undergone chondroplasty remains unknown and an answer to this question requires a randomised controlled trial with a non-operative treatment arm. It is important to note that, in general, chondroplasty is a non-specific term that encompasses several techniques for the debridement of cartilage defects.[7] The findings in this paper cannot be generalised to other types of arthroscopic and joint preservation surgery, cartilage repair and regeneration techniques, such as microfracture and autologous chondrocyte implantation.[6,31]

Our study reports the long-term outcomes following chondroplasty in a high-risk cohort of patients with cartilage damage for the first time. Our findings our stratified by a range of patient specific factors and will be important for informed consent and shared decision making between patients and clinicians. Further work is required to optimise treatment selection, however, and additional patient information may allow more accurate prediction of outcome and guide clinical management.

Conclusion

The risk of knee arthroplasty is 17 times greater in patients with a history of knee chondroplasty and in a proportion of patients, there is insufficient benefit to prevent the need for knee arthroplasty within one or two years. These important new data help inform patients and clinicians of the long-term outcomes

For peer review only

following this procedure, at the population level, for the first time. Enhanced clinical guidance on the appropriate indications for chondroplasty are required and there is a need for high-quality randomised studies to determine the relative clinical and cost effectiveness of this intervention in comparison to alternative, including non-surgical, treatments.

For peer review only

Data availability

No additional data available.

Details of contributors

SGFA: concept, methodology, analysis, writing and editing paper, guarantor.

AJRP: writing and editing paper.

AJ: methodology, analysis, editing paper.

DJB: concept, editing paper.

AJP: concept, methodology, editing paper.

Transparency declaration

The lead author (SA) affirms that the manuscript is an honest, accurate, and transparent account of the study being reported; that no important aspects of the study have been omitted; and that any discrepancies from the study as planned (and registered) have been explained.

Competing interests

Andrew Judge has received consultancy fees from Freshfields Bruckhaus Deringer (on behalf of Smith & Nephew Orthopaedics Limited), and is a member of the Data Safety and Monitoring Board (which involved receipt of fees) from Anthera Pharmaceuticals, Inc. All other authors declare no financial relationships with any organisations that might have an interest in the submitted work in the previous three years, no other relationships or activities that could appear to have influenced the submitted work.

Funding

This report is independent research supported by the National Institute for Health Research (NIHR Doctoral Research Fellowship, Mr Simon Abram, DRF-2017-10-030) and NIHR Oxford Biomedical Research Centre (BRC). Andrew Judge is supported by the NIHR Biomedical Research Centre at the University Hospitals Bristol NHS Foundation Trust and the University of Bristol. The views expressed in this publication are those of the authors and not necessarily those of the NHS, the National Institute for Health Research or the Department of Health and Social Care.

Exclusive license

The Corresponding Author has the right to grant on behalf of all authors and does grant on behalf of all authors, a worldwide licence to the Publishers and its licensees in perpetuity, in all forms, formats and media (whether known now or created in the future), to i) publish, reproduce, distribute, display and store the Contribution, ii) translate the Contribution into other languages, create adaptations, reprints, include within collections and create summaries, extracts and/or, abstracts of the Contribution and convert or allow conversion into any format including without limitation audio, iii) create any other derivative work(s) based in whole or part on the on the Contribution, iv) to exploit all subsidiary rights to exploit all subsidiary rights that currently exist or as may exist in the future in the Contribution, v) the inclusion of electronic links from the Contribution to third party material where-ever it may be located; and, vi) licence any third party to do any or all of the above.

Ethical approval

For peer review only

Not required.

For peer review only

REFERENCES

1 Järvinen TLN, Guyatt GH. Arthroscopic surgery for knee pain. *Bmj* 2016;**3934**:i3934. doi:10.1136/bmj.i3934

2 Kirkley A, Birmingham TB, Litchfield RB, *et al.* A Randomized Trial of Arthroscopic Surgery for Osteoarthritis of the Knee. *N Engl J Med* 2008;**359**:1097–107. doi:10.1056/NEJMoa0708333

3 Moseley JB, O'Malley K, Petersen NJ, *et al.* A controlled trial of arthroscopic surgery for osteoarthritis of the knee. *N Engl J Med* 2002;**347**:81–8. doi:10.1056/NEJMoa013259

4 Aaron RK, Skolnick AH, Reinert SE, *et al.* Arthroscopic Débridement for Osteoarthritis of the Knee. *J Bone Jt Surg* 2006;**88**:936–43. doi:10.2106/JBJS.D.02671

5 Palmer J. S, Monk AP, Hopewell S, *et al.* Surgical interventions for early structural knee osteoarthritis. *Cochrane Database Syst Rev* Published Online First: 23 March 2016. doi:10.1002/14651858.CD012128

6 Mistry H, Connock M, Pink J, *et al.* Autologous chondrocyte implantation in the knee: Systematic review and economic evaluation. *Health Technol Assess (Rockv)* 2017;**21**:V–160. doi:10.3310/hta21060

7 Barber FA, Iwasko NG. Treatment of Grade III Femoral Chondral Lesions: Mechanical Chondroplasty Versus Monopolar Radiofrequency Probe. *Arthrosc - J Arthrosc Relat Surg* 2006;**22**:1312–7. doi:10.1016/j.arthro.2006.06.008

8 Allen RT, Tasto JP, Cummings J, *et al.* Meniscal Debridement With an Arthroscopic Radiofrequency Wand Versus an Arthroscopic Shaver: Comparative Effects on Menisci and Underlying Articular Cartilage. *Arthrosc - J Arthrosc Relat Surg* 2006;**22**:385–93. doi:10.1016/j.arthro.2005.12.007

9 Spahn G, Kahl E, Mückley T, *et al.* Arthroscopic knee chondroplasty using a bipolar radiofrequency-based device compared to mechanical shaver: Results of a prospective, randomized, controlled study. *Knee Surgery, Sport Traumatol Arthrosc* 2008;**16**:565–73. doi:10.1007/s00167-008-0506-1

10 Lu Y, Edwards RB, Cole BJ, *et al.* Thermal chondroplasty with radiofrequency energy. An in vitro comparison of bipolar and monopolar radiofrequency devices. *Am J Sports Med* 2001;**29**:42–9.http://www.ncbi.nlm.nih.gov/pubmed/11206255

11 Dandy DJ. Abrasion Chondroplasty. *Arthrosc J Qf Artluvscopic Relat Surg*;**2**:51–3.https://ac.els-cdn.com/S0749806386800111/1-s2.0-S0749806386800111-main.pdf?_tid=b327693c-0123-4191-9c90-921c887f85db&acdnat=1522234548_babad5ec459e1423df55025bdea79d16 (accessed 28 Mar 2018).

12 National Institute for Health and Care Excellence (NICE). Arthroscopic radiofrequency chondroplasty for discrete chondral defects of the knee | Guidance and guidelines | NICE. NICE 2014. https://www.nice.org.uk/guidance/ipg493 (accessed 10 Jan 2018).

13 NHS Digital. Hospital Episode Statistics. http://content.digital.nhs.uk/hes (accessed 4 Dec 2017).

For peer review only

14 NHS Digital. *National clinical coding standards*. Stationery Office 2017.

15 NHS Digital. Hospital Episode Statistics (HES) Analysis Guide. Published Online First: 2015.http://content.digital.nhs.uk/media/1592/HES-analysis-guide/pdf/HES_Analysis_Guide_March_2015.pdf (accessed 4 Dec 2017).

16 HSCIC. Summary Hospital-level Mortality Indicator (SHMI). Indicator Specification. Version 1.25. 2017. https://www.digital.nhs.uk/SHMI

17 Charlson ME, Pompei P, Ales KL, *et al.* A new method of classifying prognostic in longitudinal studies: development and validation. J. Chronic Dis. 1987;**40**:373–83. doi:0021-9681/87

18 Zhang JX, Iwashyna TJ, Christakis NA. The performance of different lookback periods and sources of information for Charlson comorbidity adjustment in Medicare claims. *Med Care* 1999;**37**:1128–39.http://www.ncbi.nlm.nih.gov/pubmed/10549615

19 Noble M, Wright G, Smith G, *et al.* Measuring Multiple Deprivation at the Small-Area Level. *Environ Plan A Econ Sp* 2006;**38**:169–85. doi:10.1068/a37168

20 Abram SGF, Judge A, Beard DJ, *et al.* Temporal trends and regional variation in the rate of arthroscopic knee surgery in England: analysis of over 1.7 million procedures between 1997 and 2017. Has practice changed in response to new evidence? *Br J Sports Med* 2018;:bjsports-2018-099414. doi:10.1136/bjsports-2018-099414

21 Loeser RF. Age-Related Changes in the Musculoskeletal System and the Development of Osteoarthritis. *Clin Geriatr Med* 2010;**26**:371–86. doi:10.1016/j.cger.2010.03.002

22 National Institute for Health and Care Excellence. Arthroscopic knee washout, with or without debridement, for the treatment of osteoarthritis (IPG230). NICE 2007. https://www.nice.org.uk/guidance/ipg230 (accessed 8 Jan 2018).

23 Boyd JA, Gradisar IM. Total Knee Arthroplasty After Knee Arthroscopy in Patients Older Than 50 Years. *Orthopedics* 2016;**39**:1–4. doi:10.3928/01477447-20160719-01

24 Judge A, Welton NJ, Sandhu J, *et al.* Equity in access to total joint replacement of the hip and knee in England: cross sectional study. *Bmj* 2010;**341**:c4092–c4092. doi:10.1136/bmj.c4092

25 Adamson J, Ben-Shlomo Y, Chaturvedi N, *et al.* Ethnicity, socio-economic position and gender - Do they affect reported health-care seeking behaviour? *Soc Sci Med* 2003;**57**:895–904. doi:10.1016/S0277-9536(02)00458-6

26 Chaturvedi N, Rai H, Ben-Shlomo Y. Lay diagnosis and health-care-seeking behaviour for chest pain in south Asians and Europeans. *Lancet* 1997;**350**:1578–83. doi:10.1016/S0140-6736(97)06243-0

27 Jiang L, Tian W, Wang Y, *et al.* Body mass index and susceptibility to knee osteoarthritis: A systematic review and meta-analysis. *Jt Bone Spine* 2012;**79**:291–7. doi:10.1016/j.jbspin.2011.05.015

28 Khan T, Alvand A, Prieto-Alhambra D, *et al.* ACL and meniscal injuries increase the risk of primary total knee replacement for osteoarthritis: a matched case-control study using the Clinical Practice Research Datalink (CPRD). *Br J Sports Med* 2018;:bjsports-2017-097762. doi:10.1136/bjsports-2017-097762

For peer review only

29 Chan SW, Tulloch E, Cooper ES, *et al.* Montgomery and informed consent: where are we now? *BMJ* 2017;**2224**:j2224. doi:10.1136/bmj.j2224

30 UK Health Accounts 2016 - Office for National Statistics. 2016. https://www.ons.gov.uk/peoplepopulationandcommunity/healthandsocialcare/healthcaresystem/bulletins/ukhealthaccounts/2016 (accessed 5 Jun 2018).

31 Kraeutler MJ, Belk JW, Purcell JM, *et al.* Microfracture Versus Autologous Chondrocyte Implantation for Articular Cartilage Lesions in the Knee: A Systematic Review of 5-Year Outcomes. *Am J Sports Med* 2017;:036354651770191. doi:10.1177/0363546517701912

For peer review only

TABLES

TABLE 1: Demographics and descriptive statistics of cohort

	Chondroplasty Cohort		Knee Arthroplasty Cohort			
	All Cases		No Previous Chondroplasty		Previous Chondroplasty	
	**n**	**%**	**n**	**%**	**n**	**%**
**Total**	157730	100.00	568,140	94.05	35,916	5.95
**Sex**						
Male	81884	51.91	244,684	43.07	15,512	43.19
Female	75846	48.09	323,456	56.93	20,404	56.81
**Age group (years)**						
<20	2868	1.82	1,179	0.21	1	<0.01
20-39	24648	15.63	1,568	0.28	353	0.98
40-59	83258	52.79	85,797	15.1	14,023	39.04
60-79	45191	28.65	400,541	70.5	20,361	56.69
80+	1765	1.12	79,055	13.91	1,178	3.28
**Charlson comorbidity index**						
0	121605	77.10	534,399	94.06	27,331	76.1
1 - 15	34719	22.01	31,683	5.58	8,175	22.76
16 - 30	1296	0.82	1,879	0.33	393	1.09
31 - 50	110	0.07	179	0.03	17	0.05
**Index of multiple deprivation (quintiles)**						
1 = least deprived	36043	23.21	121,813	21.44	7,921	22.05
2	35189	22.66	127,672	22.47	7,938	22.1
3	32493	20.92	123,160	21.68	7,806	21.73
4	27312	17.59	103,236	18.17	6,372	17.74
5 = most deprived	24266	15.62	85,283	15.01	5,416	15.08
Missing	2427		6,976		463	
**Rurality**						
Urban	119766	76.42	423,895	74.61	27,157	75.61
Rural	36953	23.58	141,271	24.87	8,634	24.04
Missing	1011		2,974		125	
**Ethnicity**						
White	141928	94.43	525,934	92.57	34,349	95.64
Mixed	953	0.63	1,844	0.32	115	0.32
Asian	4511	3.00	19,203	3.38	804	2.24
Black	2122	1.41	5,840	1.03	193	0.54
Other	792	0.53	1,367	0.24	68	0.19
Missing	7424		13,952		387	
**Concurrent procedures**						
None	65987	41.84	-	-	-	-
Meniscal	91743	58.16	-	-	-	-

For peer review only

Table 2: Cohort demographics and adjusted odds of arthroplasty

	1-year outcome*			2-year outcome*			5-year outcome*			8-year outcome*		
	n	n TKA	% (95% CI)	n	n TKA	% (95% CI)	n	n TKA	% (95% CI)	n	n TKA	% (95% CI)
**Total**	135,197	7984	5.91% (5.78, 6.03)	114,592	10787	9.41% (9.24, 9.58)	57,267	8145	14.22% (13.94, 14.51)	16,347	2879	17.61% (17.03, 18.20)
**Sex**												
Male	69,787	3160	4.53% (4.37, 4.68)	59,101	4261	7.21% (7.00, 7.42)	29,688	3315	11.17% (10.81, 11.53)	8,514	1208	14.19% (13.45, 14.95)
Female	65,410	4824	7.38% (7.18, 7.58)	55,491	6526	11.76% (11.49, 12.03)	27,579	4830	17.51% (17.07, 17.97)	7,833	1671	21.33% (20.43, 22.26)
**Sex**												
< 20	-	-	-	-	-	-	-	-	-	-	-	-
20 - 39	21,548	48	0.22% (0.16, 0.30)	18,583	79	0.43% (0.34, 0.53)	10,004	95	0.95% (0.77, 1.16)	3,094	53	1.71% (1.29, 2.23)
40 - 59	72,345	2654	3.67% (3.53, 3.81)	60,974	4049	6.64% (6.44, 6.84)	29,844	3327	11.15% (10.79, 11.51)	8,552	1287	15.05% (14.30, 15.82)
60 - 79	39,741	4994	12.57% (12.24, 12.90)	33,680	6343	18.83% (18.42, 19.25)	16,716	4522	27.05% (26.38, 27.73)	4,508	1479	32.81% (31.44, 34.20)
80 +	1,563	288	18.43% (16.53, 20.44)	1,355	316	23.32% (21.09, 25.67)	703	201	28.59% (25.28, 32.09)	193	60	31.09% (24.64, 38.13)
**Charlson comorbidity index**												
0	104,530	5369	5.14% (5.00, 5.27)	89,081	7366	8.27% (8.09, 8.45)	45,505	5837	12.83% (12.52, 13.14)	13,362	2148	16.08% (15.46, 16.71)
1 - 15	29,475	2467	8.37% (8.06, 8.69)	24,540	3228	13.15% (12.73, 13.58)	11,371	2207	19.41% (18.69, 20.15)	2,884	701	24.31% (22.75, 25.91)
16 - 30	1,102	138	12.52% (10.63, 14.62)	898	180	20.04% (17.47, 22.82)	391	101	25.83% (21.56, 30.47)	101	30	29.70% (21.02, 39.61)
31 - 50	90	10	11.11% (5.46, 19.49)	73	13	17.81% (9.84, 28.53)	-	-	-	-	-	-
**Index of multiple deprivation (quintiles)**												
1	31,054	1846	5.94% (5.68, 6.21)	26,546	2405	9.06% (8.72, 9.41)	13,422	1835	13.67% (13.09, 14.26)	3,878	629	16.22% (15.07, 17.42)
2	30,218	1799	5.95% (5.69, 6.23)	25,638	2409	9.40% (9.04, 9.76)	12,819	1779	13.88% (13.28, 14.49)	3,474	617	17.76% (16.50, 19.07)
3	27,974	1737	6.21% (5.93, 6.50)	23,721	2324	9.80% (9.42, 10.18)	11,833	1772	14.98% (14.34, 15.63)	3,374	635	18.82% (17.51, 20.18)
4	23,312	1387	5.95% (5.65, 6.26)	19,702	1913	9.71% (9.30, 10.13)	9,771	1420	14.53% (13.84, 15.25)	2,762	496	17.96% (16.54, 19.44)
5	20,591	1104	5.36% (5.06, 5.68)	17,194	1588	9.24% (8.81, 9.68)	8,420	1197	14.22% (13.48, 14.98)	2,451	444	18.12% (16.61, 19.70)
**Rurality**												
Urban	102,665	6004	5.85% (5.71, 5.99)	86,807	8135	9.37% (9.18, 9.57)	43,287	6148	14.20% (13.88, 14.54)	2,154	12,242	17.60% (16.92, 18.28)
Rural	31,760	1944	6.12% (5.86, 6.39)	27,127	2613	9.63% (9.28, 9.99)	13,739	1980	14.41% (13.83, 15.01)	713	3,987	17.88% (16.70, 19.11)
**Ethnicity**												
White	122,261	7672	6.28% (6.14, 6.41)	103,979	10366	9.97% (9.79, 10.15)	52,267	7834	14.99% (14.68, 15.30)	14,908	2,750	18.45% (17.83, 19.08)
Mixed	750	21	2.80% (1.74, 4.25)	609	32	5.25% (3.62, 7.34)	278	22	7.91% (5.03, 11.74)	76	8	10.53% (4.66, 19.69)
Asian	3,722	130	3.49% (2.93, 4.13)	3,088	186	6.02% (5.21, 6.92)	1,465	167	11.40% (9.82, 13.14)	362	75	20.72% (16.66, 25.26)
Black	1,770	27	1.53% (1.01, 2.21)	1,466	53	3.62% (2.72, 4.70)	677	38	5.61% (4.00, 7.62)	171	15	8.77% (4.99, 14.06)
Other	645	15	2.33% (1.31, 3.81)	518	18	3.47% (2.07, 5.44)	250	21	8.40% (5.27, 12.55)	75	9	12.00% (5.64, 21.56)
**Concurrent procedures**												
None	57,208	2686	4.70% (4.52, 4.87)	50,256	3754	7.47% (7.24, 7.70)	28,578	3252	11.38% (11.01, 11.75)	9,370	1,389	14.82% (14.11, 15.56)
Meniscal	77,989	5298	6.79% (6.62, 6.97)	64,336	7033	10.93% (10.69, 11.18)	28,689	4893	17.06% (16.62, 17.50)	6,977	1,490	21.36% (20.40, 22.34)

** = excluding those patients where the date of their procedure was less than this number of years from the end of the observation period in the dataset; - = suppressed due to small numbers; CI = confidence interval; TKA = total or partial knee arthroplasty*

For peer review only

Table 3: Unadjusted and adjusted* risk of knee arthroplasty following arthroscopic chondroplasty

	**Unadjusted Risk Subsequent TKA**		**Adjusted Risk Subsequent TKA**	
	**HR**	**95% CI**	**HR**	**95% CI**
**Sex**				
Male	1.00	1.00	1.00	1.00
Female	1.61	1.57, 1.66	1.38	1.34, 1.42
**Age (per five years) ‡**				
Per year	1.35	1.35, 1.36	1.33	1.32, 1.34
**Year of treatment (per five years)**				
Year	0.99	0.96, 1.03	0.95	0.92, 0.98
**Charlson comorbidity index (per five units)**				
Charlson index	1.29	1.27, 1.31	1.03	1.01, 1.05
**Index of multiple deprivation (quintile)**				
1 = least	1.00	1.00	1.00	1.00
2	1.03	0.99, 1.08	1.07	1.03, 1.12
3	1.08	1.04, 1.13	1.17	1.12, 1.22
4	1.03	0.99, 1.08	1.20	1.15, 1.26
5 = most	1.01	0.96, 1.06	1.29	1.23, 1.36
**Rurality**				
Urban	1.00	1.00	1.00	1.00
Rural	1.03	1.00, 1.07	0.99	0.95, 1.02
**Ethnicity**				
White	1.00	1.00	1.00	1.00
Mixed	0.50	0.38, 0.65	0.66	0.51, 0.86
Asian	0.65	0.59, 0.72	0.73	0.66, 0.81
Black	0.35	0.28, 0.42	0.44	0.36, 0.54
Other	0.34	0.24, 0.48	0.45	0.32, 0.64
**Concurrent procedures**				
None	1.00	1.00	1.00	1.00
Meniscal surgery	1.52	1.48, 1.57	1.09	1.06, 1.13

** adjusted by all variables in the table; ‡ age <20 years suppressed due to small numbers; HR = hazard ratio; CI = confidence interval; TKA = total or partial knee arthroplasty*

For peer review only

Table 4: Rates and relative risk of undergoing TKA with previous chondroplasty by age at TKA in 2016-17

	**Prior chondroplasty**		**Without prior chondroplasty**		**Relative Risk**	
**Age at TKA (years)**	**Annual Rate TKA /100k**	**95% CI**	**Annual Rate TKA /100k**	**95% CI**	**RR**	**95% CI**
30 - 39	274.48 (0.27%)	190.16, 383.35 (0.19%, 0.38%)	1.60 (0.00%)	1.32, 1.92 (0.00%, 0.00%)	170.92	116.72, 250.30
40 - 49	1454.02 (1.45%)	1318.04, 1600.06 (1.32%, 1.60%)	19.79 (0.02%)	18.79, 20.82 (0.02%, 0.02%)	72.45	65.00, 80.76
50 - 59	3626.62 (3.63%)	3448.20, 3811.60 (3.45%, 3.81%)	130.68 (0.13%)	128.05, 133.35 (0.13%, 0.13%)	26.82	25.41, 28.30
60 - 69	5179.17 (5.18%)	4933.67, 5433.20 (4.93%, 5.43%)	386.68 (0.39%)	381.64, 391.77 (0.38%, 0.39%)	12.78	12.16, 13.44
70 +	6090.50 (6.09%)	5721.53, 6475.82 (5.72%, 6.48%)	520.46 (0.52%)	514.89, 526.07 (0.51%, 0.53%)	11.09	10.42, 11.80
Overall (30 +)	3494.61 (3.49%)	3394.82, 3596.52 (3.39%, 3.60%)	195.38 (0.20%)	193.90, 196.87 (0.19%, 0.20%)	17.32	16.81, 17.84

TKA = total or partial knee arthroplasty; RR = risk ratio

For peer review only

FIGURES

Figure 1: Flow chart illustrating extraction of patient level cohort

For peer review only

Figure 2: Survival curve (not undergoing knee arthroplasty) following chondroplasty by age†

† Age groups < 20 years and 80+ years suppressed due to small numbers

For peer review only

Figure 3: Survival curve (not undergoing knee arthroplasty) following chondroplasty by sex*

** Age groups < 20 years and 80+ years suppressed due to small numbers*

For peer review only

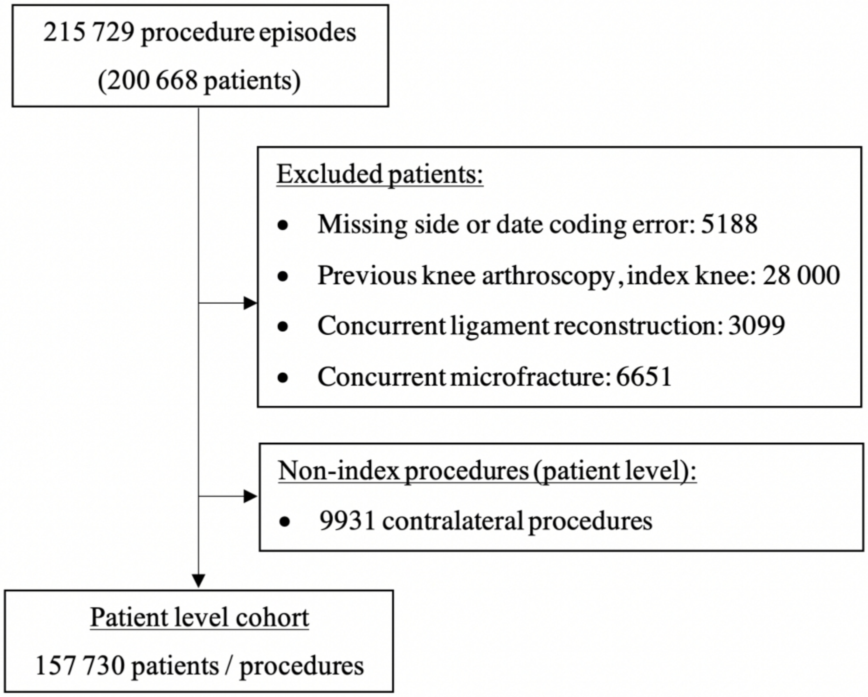

Flow chart illustrating extraction of patient level cohort

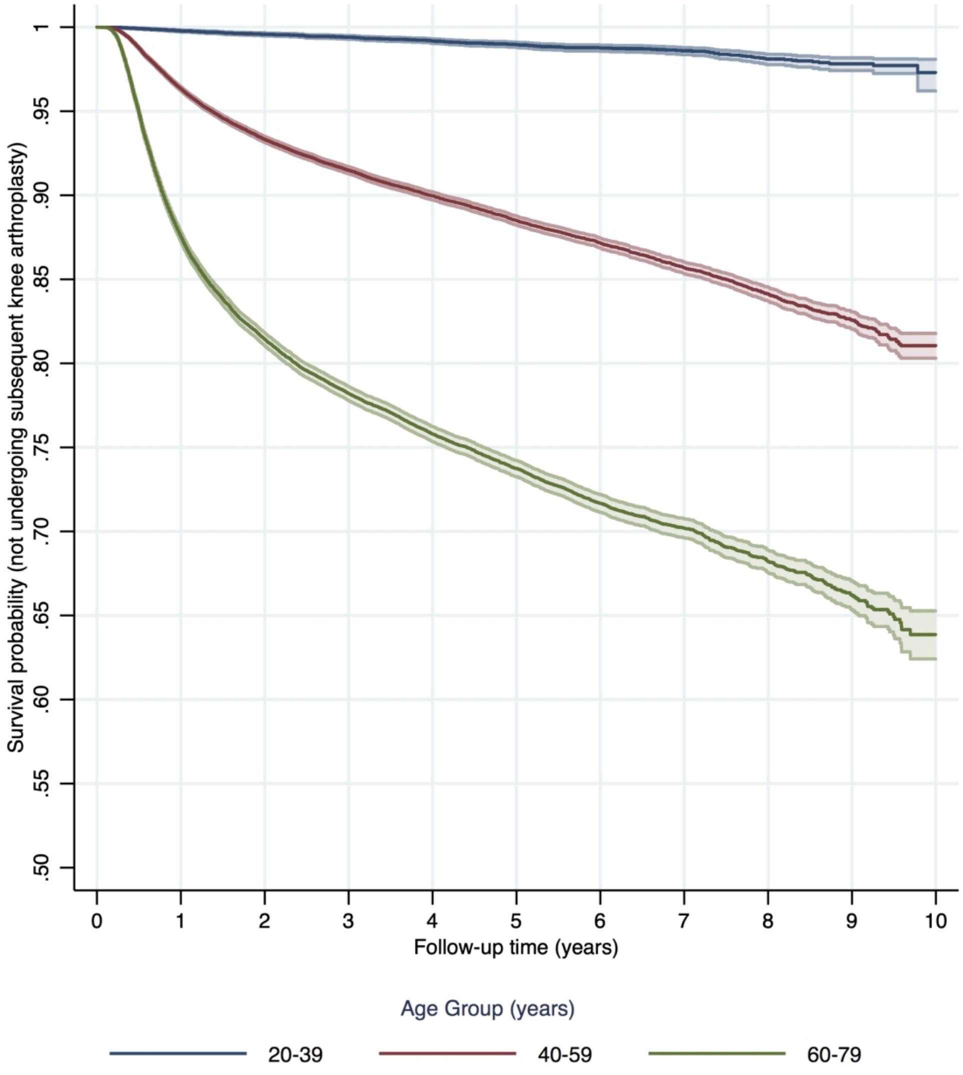

† Age groups < 20 years and 80+ years suppressed due to small numbers

Figure 2: Survival curve (not undergoing knee arthroplasty) following chondroplasty by age†

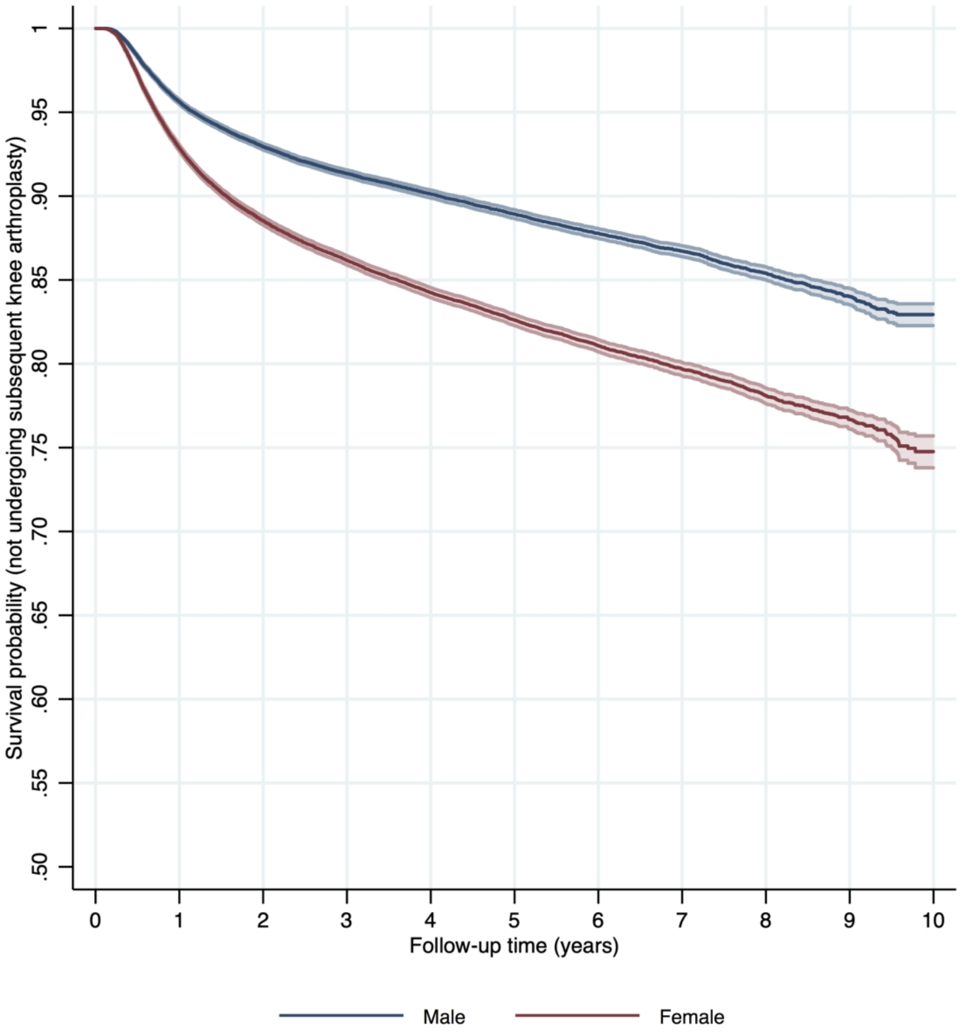

** Age groups < 20 years and 80+ years suppressed due to small numbers*

Figure 3: Survival curve (not undergoing knee arthroplasty) following chondroplasty by sex*

Appendix 1: OPCS procedure code list

PROCEDURE	OPCS 4.4	OPCS 4.5	OPCS 4.6	OPCS 4.7	Description
Chondroplasty*	W83.3*	W83.3*	W83.3*	W83.3*	Endoscopic shaving of articular cartilage
Chondroplasty*	W83.4*	W83.4*	W83.4*	W83.4*	Endoscopic articular abrasion chondroplasty
Chondroplasty*	W83.5*	W83.5*	W83.5*	W83.5*	Endoscopic articular thermal chondroplasty
Chondroplasty*	W83.6*	W83.6*	W83.6*	W83.6*	Endoscopic excision of articular cartilage NEC
Chondroplasty*	W89.1*	W89.1*	W89.1*	W89.1*	Endoscopic chondroplasty NEC
Microfracture*	W83.1*	W83.1*	W83.1*	W83.1*	Endoscopic drilling of lesion of articular cartilage
Microfracture*	W84.5*	W84.5*	W84.5*	W84.5*	Endoscopic drilling of epiphysis for repair of articular cartilage
Meniscal surgery	W82.2	W82.2	W82.2	W82.2	Endoscopic resection of semilunar cartilage NEC
Meniscal surgery	W82.3	W82.3	W82.3	W82.3	Endoscopic repair of semilunar cartilage
Meniscal surgery	W82.1	W82.1	W82.1	W82.1	Endoscopic total excision of semilunar cartilage
Ligament reconstruction (exclusion)*	W74.2	W74.2	W74.2	W74.2	Reconstruction of intra-articular ligament NEC
Ligament reconstruction (exclusion)*	W84.1	W84.1	W84.1	W84.1	Endoscopic repair of intra-articular ligament
Ligament reconstruction (exclusion)*	W84.2	W84.2	W84.2	W84.2	Endoscopic reattachment of intra-articular ligament
Ligament reconstruction (exclusion)*	W72.3	W72.3	W72.3	W72.3	Primary prosthetic replacement of intra-articular ligament
Ligament reconstruction (exclusion)*	W72.4	W72.4	W72.4	W72.4	Prosthetic replacement of intra-articular ligament NEC
Arthroplasty*	W43.1	W43.1	W43.1	W43.1	Primary total prosthetic replacement of joint using cement NEC
Arthroplasty*	W44.1	W44.1	W44.1	W44.1	Primary total prosthetic replacement of joint not using cement NEC
Arthroplasty*	W44.8	W44.8	W44.8	W44.8	Other specified total prosthetic replacement of other joint not using cement
Arthroplasty*	W44.9	W44.9	W44.9	W44.9	Unspecified total prosthetic replacement of other joint not using cement
Arthroplasty*	W45.1	W45.1	W45.1	W45.1	Primary total prosthetic replacement of joint NEC
Arthroplasty*	W45.8	W45.8	W45.8	W45.8	Other specified other total prosthetic replacement of other joint
Arthroplasty*	W45.9	W45.9	W45.9	W45.9	Unspecified other total prosthetic replacement of other joint
Arthroplasty*	W53.8	W53.8	W53.8	W53.8	Other specified prosthetic replacement of articulation of other bone not using cement
Arthroplasty	W40.1	O18.1	O18.1	O18.1	Primary hybrid prosthetic replacement of knee joint using cement
Arthroplasty	W40.8	O18.8	O18.8	O18.8	Other specified hybrid prosthetic replacement of knee joint using cement
Arthroplasty	W40.9	O18.9	O18.9	O18.9	Unspecified hybrid prosthetic replacement of knee joint using cement
Arthroplasty	W40.1	W40.1	W40.1	W40.1	Primary total prosthetic replacement of knee joint using cement
Arthroplasty	W40.8	W40.8	W40.8	W40.8	Other specified total prosthetic replacement of knee joint using cement
Arthroplasty	W40.9	W40.9	W40.9	W40.9	Unspecified total prosthetic replacement of knee joint using cement
Arthroplasty	W41.1	W41.1	W41.1	W41.1	Primary total prosthetic replacement of knee joint not using cement
Arthroplasty	W41.8	W41.8	W41.8	W41.8	Other specified total prosthetic replacement of knee joint not using cement
Arthroplasty	W41.9	W41.9	W41.9	W41.9	Unspecified total prosthetic replacement of knee joint not using cement
Arthroplasty	W42.1	W42.1	W42.1	W42.1	Primary total prosthetic replacement of knee joint NEC
Arthroplasty	W42.8	W42.8	W42.8	W42.8	Other specified other total prosthetic replacement of knee joint
Arthroplasty	W42.9	W42.9	W42.9	W42.9	Unspecified other total prosthetic replacement of knee joint
Arthroplasty*	W58.1	W58.1	W58.1	W58.1	Primary resurfacing arthroplasty of joint

**: Additional site-specific knee code required (Z846, Z765, Z845, Z844, Z774, or Z787)*

For peer review only - http://bmjopen.bmj.com/site/about/guidelines.xhtml

Appendix 2: Flow chart illustrating the extraction of the knee arthroplasty cohort

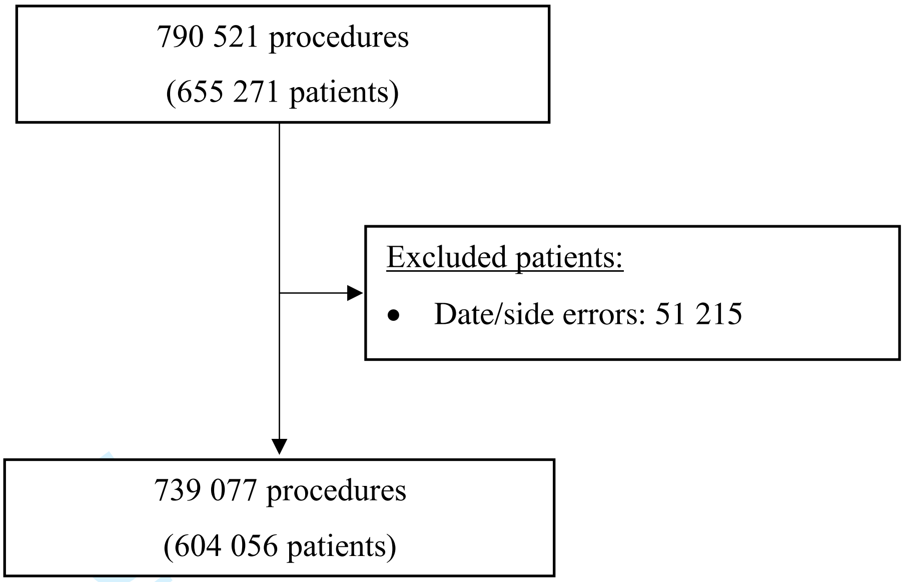

For peer review only

BMJ Open

Rates of knee arthroplasty in patients with a history of arthroscopic chondroplasty: results from a retrospective cohort study utilising the National Hospital Episode Statistics for England

Journal:	*BMJ Open*
Manuscript ID	bmjopen-2019-030609.R2
Article Type:	Original research
Date Submitted by the Author:	25-Nov-2019

Complete List of Authors:	Abram, Simon; University of Oxford Nuffield Department of Orthopaedics Rheumatology and Musculoskeletal Sciences,<br>Palmer, Antony; University of Oxford, Nuffield Department of Orthopaedics, Rheumatology and Musculoskeletal Sciences<br>Judge, Andrew; University of Bristol, Musculoskeletal Research Unit, Translational Health Sciences, Bristol Medical School, University of Bristol, Learning and Research Building, Level 1, Southmead Hospital, Southmead, BS10 5NB<br>Beard, David; University of Oxford, Nuffield Dept of Orthopaedics, Rheumatology and Musculoskeletal Sciences<br>Price, Andrew; University of Oxford, Nuffield Department of Orthopaedics, Rheumatology and Musculoskeletal Sciences

**Primary Subject Heading**:	Surgery
Secondary Subject Heading:	Rheumatology, Sports and exercise medicine, Epidemiology
Keywords:	Knee < ORTHOPAEDIC & TRAUMA SURGERY, arthroscopy, chondroplasty, knee replacement, arthroplasty, osteoarthritis

SCHOLARONE™
Manuscripts

I, the Submitting Author has the right to grant and does grant on behalf of all authors of the Work (as defined in the below author licence), an exclusive licence and/or a non-exclusive licence for contributions from authors who are: i) UK Crown employees; ii) where BMJ has agreed a CC-BY licence shall apply, and/or iii) in accordance with the terms applicable for US Federal Government officers or employees acting as part of their official duties; on a worldwide, perpetual, irrevocable, royalty-free basis to BMJ Publishing Group Ltd ("BMJ") its licensees and where the relevant Journal is co-owned by BMJ to the co-owners of the Journal, to publish the Work in this journal and any other BMJ products and to exploit all rights, as set out in our licence.

The Submitting Author accepts and understands that any supply made under these terms is made by BMJ to the Submitting Author unless you are acting as an employee on behalf of your employer or a postgraduate student of an affiliated institution which is paying any applicable article publishing charge ("APC") for Open Access articles. Where the Submitting Author wishes to make the Work available on an Open Access basis (and intends to pay the relevant APC), the terms of reuse of such Open Access shall be governed by a Creative Commons licence – details of these licences and which Creative Commons licence will apply to this Work are set out in our licence referred to above.

Other than as permitted in any relevant BMJ Author's Self Archiving Policies, I confirm this Work has not been accepted for publication elsewhere, is not being considered for publication elsewhere and does not duplicate material already published. I confirm all authors consent to publication of this Work and authorise the granting of this licence.

For peer review only

Rates of knee arthroplasty in patients with a history of arthroscopic chondroplasty: results from a retrospective cohort study utilising the National Hospital Episode Statistics for England

1. S.G.F. Abram, MRCS[1,2]
2. A.J.R. Palmer, DPhil[1,2]
3. A. Judge, PhD[1,3,4]
4. D.J. Beard, DPhil[1,2]
5. A.J. Price, DPhil[1,2]

Author affiliations:

[1]Nuffield Department of Orthopaedics, Rheumatology and Musculoskeletal Sciences, University of Oxford
[2]NIHR Biomedical Research Centre, Oxford
[3]Musculoskeletal Research Unit, University of Bristol
[4]NIHR Biomedical Research Centre, Bristol

Author titles:

SGFA: NIHR Doctoral Research Fellow
AJRP: NIHR Clinical Lecturer
AJ: Professor of Translational Statistics
DJB: Professor of Musculoskeletal Sciences
AJP: Professor of Orthopaedics

Corresponding author:

Simon Abram

E: simon.abram@ndorms.ox.ac.uk

T: 01865 223425

Botnar Research Centre, University of Oxford

Nuffield Department of Orthopaedics, Rheumatology and Musculoskeletal Sciences

Windmill Road, Oxford OX3 7LD. UK

Keywords

knee; arthroscopy; chondroplasty; knee replacement; arthroplasty; outcomes; osteoarthritis

Word count

2694

For peer review only

ABSTRACT

OBJECTIVE

The purpose of this study was to analyse the rate of knee arthroplasty in the population of patients with a history of arthroscopic chondroplasty of the knee, in England, over ten years, with comparison to general population data for patients without a history of chondroplasty.

DESIGN

Retrospective cohort study

SETTING

English hospital episode statistics (HES) data

PARTICIPANTS AND INTERVENTIONS

Patients undergoing arthroscopic chondroplasty in England between 2007/08 and 2016/17 were identified. Patients undergoing previous arthroscopic knee surgery or simultaneous cruciate ligament reconstruction or microfracture in the same knee were excluded.

OUTCOMES

Patients subsequently undergoing a knee arthroplasty in the same knee were identified and mortality-adjusted survival analysis was performed (survival without undergoing knee arthroplasty). A Cox proportional hazards model was used to identify factors associated with knee arthroplasty. Relative risk of knee arthroplasty (total or partial) in comparison to the general population was determined.

RESULTS

Through 2007-17, 157,730 eligible chondroplasty patients were identified. Within one-year, 5.91% (7984/135197; 95% CI 5.78 to 6.03) underwent knee arthroplasty and 14.22% (8145/57267; 95% CI 13.94 to 14.51) within five-years. Patients aged over 30 years with a history of chondroplasty were 17.32 times (risk ratio; 95% CI 16.81 to 17.84) more likely to undergo arthroplasty than the general population without a history of chondroplasty.

CONCLUSIONS

Patients with cartilage lesions of the knee, treated with arthroscopic chondroplasty, are at greater risk of subsequent knee arthroplasty than the general population and for a proportion of patients, there is insufficient benefit to prevent the need for knee arthroplasty within one- to five-years. These important new data will inform patients of the anticipated outcomes following this procedure. The risk in comparison to non-operative treatment remains unknown and there is an urgent need for a randomised clinical trial in this population.

For peer review only

STRENGTHS AND LIMITATIONS OF THIS STUDY

- Strengths of the data source analysed in this study include comprehensive, national, data collection and the ability to match treatment with outcomes, including by the laterality of intervention, over time.
- This is the largest cohort of patients undergoing arthroscopic chondroplasty that has been reported, with strict inclusion criteria, excluding patients with a history of previous surgery to the same knee and those undergoing simultaneous ligament reconstruction or microfracture.
- All studies of this design rely on coding accuracy and some coding errors are inevitable; although outcomes were stratified by a range of patient factors, unmeasured potential confounders include body mass index, limb alignment, baseline radiographic status.
- Knee arthroplasty is an end-stage outcome and will underestimate the true burden and severity of symptomatic osteoarthritis in this population.
- The outcome had these patients not undergone arthroscopic chondroplasty remains unknown.

For peer review only

INTRODUCTION

Around 2 million knee arthroscopy procedures are performed worldwide each year.[1] Historically, knee washout and 'debridement' was shown to be ineffective for the treatment of advanced osteoarthritis.[2–4] For early osteoarthritis, however, a number of surgical and non-surgical treatments are available and treatment selection is challenging.[5] The aim of treatment in these cases is to improve symptoms and delay or prevent progressive osteoarthritis.[6]

Chondroplasty is a non-specific term that encompasses several techniques for the treatment of cartilage defects.[7] It includes debridement and abrasion using mechanical 'shavers' and, more recently, thermal or radiofrequency techniques have also emerged despite some concerns these techniques might risk inducing localised chondrocyte death.[7–11] Recent national guidance was cautiously supportive of radiofrequency chondroplasty for the treatment of "discrete chondral defects" based on a small number of clinical trials comparing the outcomes of mechanical and radiofrequency techniques.[12] It is not known which patients are most likely to benefit from chondroplasty procedures and when the procedure does not provide sustained benefit, knee arthroplasty is often indicated. The success rate of chondroplasty is, however, poorly understood and the proportion of patients undergoing subsequent knee arthroplasty after this intervention has been unknown.

The purpose of this study was to determine the proportion of patients undergoing knee chondroplasty procedures that subsequently receive a knee arthroplasty in the same knee, with specific focus on the proportion of patients undergoing early arthroplasty with one-year or two-years of chondroplasty. Factors associated with the risk of subsequent arthroplasty are reported and the relative risk in comparison to the general population determined.

For peer review only

METHODS

Data source

National Hospital Episode Statistics (HES) data was obtained (application DARS-NIC-68703) in a deidentified (pseudo-anonymised) format from NHS Digital.[13] HES contains a record of deidentified patient attendances at National Health Service (NHS) hospitals in England.[13] The data is submitted by hospitals to claim payment for the services they provide and is also intended for secondary use, including research. HES includes episodes of care delivered in treatment centres (including those in the independent sector) funded by the NHS, episodes of care in England where patients are resident outside of England, and privately funded patients treated within NHS England hospitals. The information recorded in the HES database includes patient demographic and residence data, primary and secondary diagnoses including comorbidities, and all procedures undertaken.

Procedures

All HES records between 1 April 2007 and 31 March 2017 were extracted for patients undergoing arthroscopic chondroplasty. Patients undergoing previous arthroscopic knee surgery or simultaneous cruciate ligament reconstruction or microfracture in the same knee were excluded. Procedures were identified using the Classification of Surgical Operations and Procedures (OPCS-4) codes recorded within the HES data (see appendix 1 for OPCS-4 code list).[14] All knee arthroplasty (partial or total) procedures were also identified (appendix 2) for the whole population to enable the relative risk of knee arthroplasty with and without a history of chondroplasty to be determined.

Outcomes

The primary outcome was knee arthroplasty, matched to the side of any previous chondroplasty (using recorded OPCS-4 laterality codes).

Statistical analysis

Stata v15.1 (StataCorp, College Station, Texas, USA) was used to perform all analysis. In accordance with Office for National Statistics (ONS) and NHS Digital guidance, rates where the number of events was less than six were suppressed.[15] Procedures with date errors or missing laterality were excluded. The absolute rate of knee arthroplasty was determined at 1 year, 2 years, 5 years, and 8 years following arthroscopic chondroplasty as the proportion of the cohort with this minimum period of follow-up. Mortality adjusted Kaplan-Meier survival analysis (survival was defined as not undergoing knee arthroplasty) was also performed and stratified by patient age group and sex.

A Cox proportional-hazards model was used first to calculate the unadjusted hazard ratio of knee arthroplasty over time by age group, sex, index of multiple deprivation (quintile derived from regional

For peer review only

factors in England including average income, employment, education, housing, and crime; 1=least deprived area, 5=most deprived), ethnicity, modified Charlson comorbidity index (derived with maximum 5-year diagnosis code lookback period),[16–18] year of treatment (chondroplasty), rurality, and ethnicity respectively.[16–19] The hazard ratios were then adjusted including all these variables in the model.

The relative risk (risk ratio) of knee arthroplasty in the population of patients with a history of chondroplasty in comparison to the general population (without a history of chondroplasty) was estimated for the year 2016-17. All patients undergoing knee arthroplasty in 2016-17 were identified and the number of these patients with a recorded previous chondroplasty (in the prior 10-years of HES data), versus those without, made up the numerator for each respective population. The chondroplasty population denominator was the number of patients with a history of chondroplasty that had not undergone a knee arthroplasty prior to 2016-17. The denominator for the non-chondroplasty population was the ONS mid-year population estimate less the chondroplasty population.

Patient and public involvement

There was no patient and public involvement in this study.

For peer review only

RESULTS

Over the study period, 157,730 chondroplasty patients were identified as eligible for analysis (Figure 1). The mean age of the chondroplasty cohort was 51.7 year (SD 13.8) and 48.1% were female (Table 1). Over the same period, 604,056 patients underwent knee arthroplasty, of which 35,916 (5.95%) had a record of a previous chondroplasty (Table 1).

Overall, following chondroplasty, 5.91% (7984/135197; 95% confidence interval [CI] 5.78 to 6.03) patients underwent knee arthroplasty within 1 year, 9.41% (10787/114592; 95% CI 9.24 to 9.58) within 2 years, 14.22% (8145/57267; 95% CI 13.94 to 14.51) within 5 years, and 17.61% (2879/16347; 95% CI 17.03 to 18.20) within 8 years (Table 2). The risk of arthroplasty was greater in female patients (adjusted hazard ratio [HR] 1.38; 95% CI 1.34 to 1.42) and in older patients (adjusted HR 1.33 per five years; 95% CI 1.32 to 1.34) (Table 3, Figure 2; Figure 3). Patients with a greater comorbidity index were also at increased risk of subsequently undergoing arthroplasty (adjusted HR 1.03 per five units Charlson index; 95% CI 1.01 to 1.05).

The risk of knee arthroplasty after chondroplasty fell slightly over time, by year of chondroplasty treatment (adjusted HR 0.95 per five years; 95% CI 0.92 to 0.98). Patients in regions of increased deprivation and patients of white ethnicity were at greater risk of subsequent arthroplasty (Table 2). Patients undergoing concurrent meniscal surgery were also at greater risk of subsequent arthroplasty (adjusted HR 1.09; 95% CI 1.06 to 1.13).

In 2016-17, the rate of knee arthroplasty was 3.49% (95% CI 3.39 to 3.60) in patients (aged 30 or older) with a recorded history of chondroplasty and 0.20% (95% CI 0.19 to 0.20) in patients without a record of chondroplasty. This corresponded to an overall relative risk of knee arthroplasty for the chondroplasty cohort patients of 17.32 times (risk ratio [RR]; 95% CI 16.81 to 17.84) that of the general population (Table 4).

Although the absolute annual rate of knee arthroplasty was low, the relative risk of undergoing knee arthroplasty at a younger age was greatly elevated in comparison to arthroplasty at an older age, as shown in Table 4. Patients aged 30-39 with a history of a previous chondroplasty were 170.92 times (RR; 95% CI 116.72 to 250.30) more likely to undergo knee arthroplasty than the general population, per year, in comparison to 11.09 times (RR; 95% CI 10.42 to 11.80) more likely for the over 69 age group.

For peer review only

DISCUSSION

Principal findings

Patients undergoing chondroplasty procedures of the knee have a 17 times increased risk of receiving a knee arthroplasty compared with the general population. Nearly 10% of patients will have received a knee arthroplasty within two years of the chondroplasty procedure. The relative risk of undergoing arthroplasty at a young age is particularly elevated, reaching 171 times the general population rate for arthroplasty between the ages of 30 and 39 years of age. For a proportion of patients, the results indicate insufficient benefit to prevent the need for knee arthroplasty within one or two years, but the risk had these patients not undergone chondroplasty remains unknown.

Comparison to other studies

We previously reported trends in chondroplasty surgery in England, but data from other countries is not available.[20] The age-sex standardised rate of chondroplasty increased 191% from 17.6/100,000 (95% CI 17.2 to 18.0) in 2007/08 to 51.2/100,000 (95% CI 50.6 to 51.7) in 2016/17.[20] The rate of chondroplasty was greatest in patients aged 40-59 years (increasing 210% from 34.3/100,000 in 2007/08 to 106.4/100,000 in 2016/17.[20]

In England, although national guidance has been cautiously supportive of radiofrequency chondroplasty for specific indications, there is only limited evidence demonstrating the effectiveness of chondroplasty compared with alternative surgical or non-surgical treatments.[12] The only randomised studies have been limited to comparisons of different chondroplasty techniques.[10,12] Long-term outcomes following chondroplasty have yet to be reported.[12]

Older patients are much more likely to have generalised osteoarthritis, rather than "discrete chondral defects" for which the national guidance supports radiofrequency chondroplasty.[12,21] For more generalised osteoarthritis, chondroplasty is analogous to debridement and washout, where multiple clinical trials demonstrate no benefit.[2–4] The use of chondroplasty in the treatment of patients with more generalised chondral pathology is therefore unproven and not recommended.[22] In our study, there was considerable age-group variation in outcomes, with 18.8% of patients aged 60-79-years undergoing arthroplasty within two-years of chondroplasty, in comparison to 0.43% for patients undergoing chondroplasty aged 20-39-years. This observation is consistent with the presence of more established osteoarthritis in older age groups.

Female patients were observed to be of greater risk of subsequent arthroplasty in our study. This has previously been observed following knee arthroscopy in the United States.[23] Patients of white ethnicity and greater deprivation were also at greater risk in our cohort. These findings may reflect differences in

For peer review only

healthcare access including treatment thresholds for either the chondroplasty or knee arthroplasty, or differences in care seeking behaviour which has been shown to be influenced by socioeconomic, cultural, occupational, and psychological factors, or there could be biological factors underlying the observation.[24–26] Patients with a greater comorbidity index were more likely to undergo subsequent arthroplasty, and the reason for this is unclear. One possible explanation might be an association between comorbidity and higher body mass index (BMI), which is not recorded in this dataset, with patients having a greater BMI being more likely to progress to end-stage osteoarthritis, or that these patients had more severe pathology at the time of their index chondroplasty.[27] Patients undergoing concurrent meniscal surgery were also more likely to undergo subsequent arthroplasty, which is expected given the association between meniscal injury, osteoarthritis, and knee arthroplasty.[28]

Recently, there has been renewed focus on the importance of and requirements for individualised patient consent.[29] Our findings make an important contribution to the current evidence, and patients can now be appropriately counselled and consented with knowledge of anticipated long-term outcomes.

For peer review only

Strength and limitations

A key strength of our study is the identification of all knee chondroplasty procedures performed in the National Health Service over a 10-year period, creating the largest reported cohort of patients receiving this procedure. Patients with a history of prior arthroscopy in the same knee, simultaneous ligament reconstruction or microfracture were excluded as potential confounding factors. It should still be noted that patients undergoing non-NHS treatment, for example knee arthroplasty in the private sector after a previous knee arthroscopy under NHS care, would not be captured in this dataset and the number of these procedures performed in the private sector is currently unknown. National data does indicate, however, that private healthcare expenditure as a proportion of total healthcare expenditure has remained relatively stable at around 17-18% of total health expenditure between 2005 and 2015.[30] For all observational studies utilising large datasets there may be some concerns raised about coding accuracy. The data in our study was cleaned prior to analysis, excluding patients where procedures were missing the side of intervention, and cases where date coding errors were identified. Although some other data coding errors are inevitable, data errors in procedure coding would result in hospitals not receiving payment for surgery performed, and this provides a strong incentive for data accuracy with regards to the coding data analysed in this study.

We were able to stratify risk of arthroplasty by a large number of patient factors, but certain procedure specific data is not recorded. Operative factors, such as the affected compartment of the knee and extent of initial cartilage damage before intervention, are not recorded in this database. These factors may be important in determining outcome, for example, there are likely to be differences in long-term outcomes between chondroplasty performed to the tibiofemoral joint in comparison to the patellofemoral joint.[31] Other unmeasured sources of potential confounding include BMI, leg alignment, and radiographic status at

the time of intervention. These are important considerations when considering if a patient is suitable for chondral surgery intervention, but the specific impact of these factors on long-term outcomes in this population remains uncertain. Subjective, patient-reported, symptomatic outcome data is not yet available for this cohort and radiographic outcomes are not recorded in the HES database. Instead, our study focussed on the objective, measurable outcome of knee arthroplasty, matched to the same knee as the previous chondroplasty surgery intervention. Although knee arthroplasty represents the end-stage of symptomatic failure for patients with osteoarthritis, it is likely to considerably underestimate the overall health and symptom burden in this cohort. Patients, particularly younger patients, may not have been willing or suitable candidates for knee arthroplasty, and the threshold for arthroplasty may have been much higher for younger age groups or older patients with multiple comorbidities. It is also important to note that, in general, "chondroplasty" is a non-specific term that encompasses several techniques for the debridement of cartilage defects.[7] The findings in this paper cannot be generalised to other types of arthroscopic and joint preservation surgery, cartilage repair and regeneration techniques, such as microfracture and autologous chondrocyte implantation.[6,31]

Our study represents a high-risk cohort of patients with cartilage damage. It is unknown from this observational data whether undergoing the chondroplasty procedure was beneficial to the symptoms or prognosis of these individuals over the full study period. That is, it is not known whether the chondroplasty procedure delayed or prevented arthroplasty in those patients that did not undergo arthroplasty (approximately 86% by 5-years), in which case delivery of the intervention may have been cost-effective, or the converse interpretation is that the procedure may have been over-used and that the natural history of symptomatic osteoarthritis in this population was unaltered. For example, the observed proportion of patients undergoing arthroplasty within 1-year of their arthroscopic chondroplasty (6%) is suggestive of sub-optimal treatment selection. These individuals are highly unlikely to have had only localised or partial thickness lesions and our results may indicate that knee arthroplasty may have been a more appropriate treatment. Nevertheless, the symptomatic outcome in the patients that did not undergo arthroplasty is not known and the answer to whether the procedure is cost-effective with optimal patient selection is unknown and requires evaluation in a high-quality randomised controlled trial with a non-operative treatment arm. Such a trial should help to evaluate the optimal indications for chondroplasty, assess the relative rate of progression of treated chondral damage with versus without chondroplasty, and ultimately determine whether appropriate use of chondroplasty is beneficial to patient outcome including, potentially, the long-term demand for knee arthroplasty.

Our study reports the long-term outcomes following chondroplasty in a high-risk cohort of patients with cartilage damage for the first time. Our findings our stratified by a range of patient specific factors however further work is required to optimise treatment selection and additional patient information may allow more accurate prediction of outcome and guide clinical management.

For peer review only

Conclusion

The risk of knee arthroplasty is 17 times greater in patients with a history of knee chondroplasty and in a proportion of patients, there is insufficient benefit to prevent the need for knee arthroplasty within one or two years. These important new data help inform patients and clinicians of the long-term outcomes following this procedure, at the population level, for the first time. Enhanced clinical guidance on the appropriate indications for chondroplasty are required and there is a need for high-quality randomised studies to determine the relative clinical and cost effectiveness of this intervention in comparison to alternative, including non-surgical, treatments.

For peer review only

Data availability

No additional data available.

Details of contributors

SGFA: concept, methodology, analysis, writing and editing paper, guarantor.

AJRP: writing and editing paper.

AJ: methodology, analysis, editing paper.

DJB: concept, editing paper.

AJP: concept, methodology, editing paper.

Transparency declaration

The lead author (SA) affirms that the manuscript is an honest, accurate, and transparent account of the study being reported; that no important aspects of the study have been omitted; and that any discrepancies from the study as planned (and registered) have been explained.

Competing interests

Andrew Judge has received consultancy fees from Freshfields Bruckhaus Deringer (on behalf of Smith & Nephew Orthopaedics Limited), and is a member of the Data Safety and Monitoring Board (which involved receipt of fees) from Anthera Pharmaceuticals, Inc. All other authors declare no financial relationships with any organisations that might have an interest in the submitted work in the previous three years, no other relationships or activities that could appear to have influenced the submitted work.

Funding

This report is independent research supported by the National Institute for Health Research (NIHR Doctoral Research Fellowship, Mr Simon Abram, DRF-2017-10-030) and NIHR Oxford Biomedical Research Centre (BRC). Andrew Judge is supported by the NIHR Biomedical Research Centre at the University Hospitals Bristol NHS Foundation Trust and the University of Bristol. The views expressed in this publication are those of the authors and not necessarily those of the NHS, the National Institute for Health Research or the Department of Health and Social Care.

Exclusive license

The Corresponding Author has the right to grant on behalf of all authors and does grant on behalf of all authors, a worldwide licence to the Publishers and its licensees in perpetuity, in all forms, formats and media (whether known now or created in the future), to i) publish, reproduce, distribute, display and store the Contribution, ii) translate the Contribution into other languages, create adaptations, reprints, include within collections and create summaries, extracts and/or, abstracts of the Contribution and convert or allow conversion into any format including without limitation audio, iii) create any other derivative work(s) based in whole or part on the on the Contribution, iv) to exploit all subsidiary rights to exploit all subsidiary rights that currently exist or as may exist in the future in the Contribution, v) the inclusion of electronic links from the Contribution to third party material where-ever it may be located; and, vi) licence any third party to do any or all of the above.

Ethical approval

For peer review only

Not required.

For peer review only

REFERENCES

1 Järvinen TLN, Guyatt GH. Arthroscopic surgery for knee pain. *Bmj* 2016;**3934**:i3934. doi:10.1136/bmj.i3934

2 Kirkley A, Birmingham TB, Litchfield RB, *et al.* A Randomized Trial of Arthroscopic Surgery for Osteoarthritis of the Knee. *N Engl J Med* 2008;**359**:1097–107. doi:10.1056/NEJMoa0708333

3 Moseley JB, O'Malley K, Petersen NJ, *et al.* A controlled trial of arthroscopic surgery for osteoarthritis of the knee. *N Engl J Med* 2002;**347**:81–8. doi:10.1056/NEJMoa013259

4 Aaron RK, Skolnick AH, Reinert SE, *et al.* Arthroscopic Débridement for Osteoarthritis of the Knee. *J Bone Jt Surg* 2006;**88**:936–43. doi:10.2106/JBJS.D.02671

5 Palmer J. S, Monk AP, Hopewell S, *et al.* Surgical interventions for early structural knee osteoarthritis. *Cochrane Database Syst Rev* Published Online First: 23 March 2016. doi:10.1002/14651858.CD012128

6 Mistry H, Connock M, Pink J, *et al.* Autologous chondrocyte implantation in the knee: Systematic review and economic evaluation. *Health Technol Assess (Rockv)* 2017;**21**:V–160. doi:10.3310/hta21060

7 Barber FA, Iwasko NG. Treatment of Grade III Femoral Chondral Lesions: Mechanical Chondroplasty Versus Monopolar Radiofrequency Probe. *Arthrosc - J Arthrosc Relat Surg* 2006;**22**:1312–7. doi:10.1016/j.arthro.2006.06.008

8 Allen RT, Tasto JP, Cummings J, *et al.* Meniscal Debridement With an Arthroscopic Radiofrequency Wand Versus an Arthroscopic Shaver: Comparative Effects on Menisci and Underlying Articular Cartilage. *Arthrosc - J Arthrosc Relat Surg* 2006;**22**:385–93. doi:10.1016/j.arthro.2005.12.007

9 Spahn G, Kahl E, Mückley T, *et al.* Arthroscopic knee chondroplasty using a bipolar radiofrequency-based device compared to mechanical shaver: Results of a prospective, randomized, controlled study. *Knee Surgery, Sport Traumatol Arthrosc* 2008;**16**:565–73. doi:10.1007/s00167-008-0506-1

10 Lu Y, Edwards RB, Cole BJ, *et al.* Thermal chondroplasty with radiofrequency energy. An in vitro comparison of bipolar and monopolar radiofrequency devices. *Am J Sports Med* 2001;**29**:42–9.http://www.ncbi.nlm.nih.gov/pubmed/11206255

11 Dandy DJ. Abrasion Chondroplasty. *Arthrosc J Qf Artluvscopic Relat Surg*;**2**:51–3.https://ac.els-cdn.com/S0749806386800111/1-s2.0-S0749806386800111-main.pdf?_tid=b327693c-0123-4191-9c90-921c887f85db&acdnat=1522234548_babad5ec459e1423df55025bdea79d16 (accessed 28 Mar 2018).

12 National Institute for Health and Care Excellence (NICE). Arthroscopic radiofrequency chondroplasty for discrete chondral defects of the knee | Guidance and guidelines | NICE. NICE 2014. https://www.nice.org.uk/guidance/ipg493 (accessed 10 Jan 2018).

13 NHS Digital. Hospital Episode Statistics. http://content.digital.nhs.uk/hes (accessed 4 Dec 2017).

For peer review only

14 NHS Digital. *National clinical coding standards*. Stationery Office 2017.

15 NHS Digital. Hospital Episode Statistics (HES) Analysis Guide. Published Online First: 2015.http://content.digital.nhs.uk/media/1592/HES-analysis-guide/pdf/HES_Analysis_Guide_March_2015.pdf (accessed 4 Dec 2017).

16 HSCIC. Summary Hospital-level Mortality Indicator (SHMI). Indicator Specification. Version 1.25. 2017. https://www.digital.nhs.uk/SHMI

17 Charlson ME, Pompei P, Ales KL, *et al.* A new method of classifying prognostic in longitudinal studies: development and validation. J. Chronic Dis. 1987;**40**:373–83. doi:0021-9681/87

18 Zhang JX, Iwashyna TJ, Christakis NA. The performance of different lookback periods and sources of information for Charlson comorbidity adjustment in Medicare claims. *Med Care* 1999;**37**:1128–39.http://www.ncbi.nlm.nih.gov/pubmed/10549615

19 Noble M, Wright G, Smith G, *et al.* Measuring Multiple Deprivation at the Small-Area Level. *Environ Plan A Econ Sp* 2006;**38**:169–85. doi:10.1068/a37168

20 Abram SGF, Judge A, Beard DJ, *et al.* Temporal trends and regional variation in the rate of arthroscopic knee surgery in England: analysis of over 1.7 million procedures between 1997 and 2017. Has practice changed in response to new evidence? *Br J Sports Med* 2018;:bjsports-2018-099414. doi:10.1136/bjsports-2018-099414

21 Loeser RF. Age-Related Changes in the Musculoskeletal System and the Development of Osteoarthritis. *Clin Geriatr Med* 2010;**26**:371–86. doi:10.1016/j.cger.2010.03.002

22 National Institute for Health and Care Excellence. Arthroscopic knee washout, with or without debridement, for the treatment of osteoarthritis (IPG230). NICE 2007. https://www.nice.org.uk/guidance/ipg230 (accessed 8 Jan 2018).

23 Boyd JA, Gradisar IM. Total Knee Arthroplasty After Knee Arthroscopy in Patients Older Than 50 Years. *Orthopedics* 2016;**39**:1–4. doi:10.3928/01477447-20160719-01

24 Judge A, Welton NJ, Sandhu J, *et al.* Equity in access to total joint replacement of the hip and knee in England: cross sectional study. *Bmj* 2010;**341**:c4092–c4092. doi:10.1136/bmj.c4092

25 Adamson J, Ben-Shlomo Y, Chaturvedi N, *et al.* Ethnicity, socio-economic position and gender - Do they affect reported health-care seeking behaviour? *Soc Sci Med* 2003;**57**:895–904. doi:10.1016/S0277-9536(02)00458-6

26 Chaturvedi N, Rai H, Ben-Shlomo Y. Lay diagnosis and health-care-seeking behaviour for chest pain in south Asians and Europeans. *Lancet* 1997;**350**:1578–83. doi:10.1016/S0140-6736(97)06243-0

27 Jiang L, Tian W, Wang Y, *et al.* Body mass index and susceptibility to knee osteoarthritis: A systematic review and meta-analysis. *Jt Bone Spine* 2012;**79**:291–7. doi:10.1016/j.jbspin.2011.05.015

28 Khan T, Alvand A, Prieto-Alhambra D, *et al.* ACL and meniscal injuries increase the risk of primary total knee replacement for osteoarthritis: a matched case-control study using the Clinical Practice Research Datalink (CPRD). *Br J Sports Med* 2018;:bjsports-2017-097762. doi:10.1136/bjsports-2017-097762

For peer review only

29 Chan SW, Tulloch E, Cooper ES, *et al.* Montgomery and informed consent: where are we now? *BMJ* 2017;**2224**:j2224. doi:10.1136/bmj.j2224

30 UK Health Accounts 2016 - Office for National Statistics. 2016. https://www.ons.gov.uk/peoplepopulationandcommunity/healthandsocialcare/healthcaresystem/bulletins/ukhealthaccounts/2016 (accessed 5 Jun 2018).

31 Kraeutler MJ, Belk JW, Purcell JM, *et al.* Microfracture Versus Autologous Chondrocyte Implantation for Articular Cartilage Lesions in the Knee: A Systematic Review of 5-Year Outcomes. *Am J Sports Med* 2017;:036354651770191. doi:10.1177/0363546517701912

For peer review only

TABLES

TABLE 1: Demographics and descriptive statistics of cohort

	Chondroplasty Cohort		Knee Arthroplasty Cohort			
	All Cases		No Previous Chondroplasty		Previous Chondroplasty	
	**n**	**%**	**n**	**%**	**n**	**%**
**Total**	157730	100.00	568,140	94.05	35,916	5.95
**Sex**						
Male	81884	51.91	244,684	43.07	15,512	43.19
Female	75846	48.09	323,456	56.93	20,404	56.81
**Age group (years)**						
<20	2868	1.82	1,179	0.21	1	<0.01
20-39	24648	15.63	1,568	0.28	353	0.98
40-59	83258	52.79	85,797	15.1	14,023	39.04
60-79	45191	28.65	400,541	70.5	20,361	56.69
80+	1765	1.12	79,055	13.91	1,178	3.28
**Charlson comorbidity index**						
0	121605	77.10	534,399	94.06	27,331	76.1
1 - 15	34719	22.01	31,683	5.58	8,175	22.76
16 - 30	1296	0.82	1,879	0.33	393	1.09
31 - 50	110	0.07	179	0.03	17	0.05
**Index of multiple deprivation (quintiles)**						
1 = least deprived	36043	23.21	121,813	21.44	7,921	22.05
2	35189	22.66	127,672	22.47	7,938	22.1
3	32493	20.92	123,160	21.68	7,806	21.73
4	27312	17.59	103,236	18.17	6,372	17.74
5 = most deprived	24266	15.62	85,283	15.01	5,416	15.08
Missing	2427		6,976		463	
**Rurality**						
Urban	119766	76.42	423,895	74.61	27,157	75.61
Rural	36953	23.58	141,271	24.87	8,634	24.04
Missing	1011		2,974		125	
**Ethnicity**						
White	141928	94.43	525,934	92.57	34,349	95.64
Mixed	953	0.63	1,844	0.32	115	0.32
Asian	4511	3.00	19,203	3.38	804	2.24
Black	2122	1.41	5,840	1.03	193	0.54
Other	792	0.53	1,367	0.24	68	0.19
Missing	7424		13,952		387	
**Concurrent procedures**						
None	65987	41.84	-	-	-	-
Meniscal	91743	58.16	-	-	-	-

For peer review only

Table 2: Cohort demographics and adjusted odds of arthroplasty

	1-year outcome*			2-year outcome*			5-year outcome*			8-year outcome*		
	n	n TKA	% (95% CI)	n	n TKA	% (95% CI)	n	n TKA	% (95% CI)	n	n TKA	% (95% CI)
**Total**	135,197	7984	5.91% (5.78, 6.03)	114,592	10787	9.41% (9.24, 9.58)	57,267	8145	14.22% (13.94, 14.51)	16,347	2879	17.61% (17.03, 18.20)
**Sex**												
Male	69,787	3160	4.53% (4.37, 4.68)	59,101	4261	7.21% (7.00, 7.42)	29,688	3315	11.17% (10.81, 11.53)	8,514	1208	14.19% (13.45, 14.95)
Female	65,410	4824	7.38% (7.18, 7.58)	55,491	6526	11.76% (11.49, 12.03)	27,579	4830	17.51% (17.07, 17.97)	7,833	1671	21.33% (20.43, 22.26)
**Sex**												
< 20	-	-	-	-	-	-	-	-	-	-	-	-
20 - 39	21,548	48	0.22% (0.16, 0.30)	18,583	79	0.43% (0.34, 0.53)	10,004	95	0.95% (0.77, 1.16)	3,094	53	1.71% (1.29, 2.23)
40 - 59	72,345	2654	3.67% (3.53, 3.81)	60,974	4049	6.64% (6.44, 6.84)	29,844	3327	11.15% (10.79, 11.51)	8,552	1287	15.05% (14.30, 15.82)
60 - 79	39,741	4994	12.57% (12.24, 12.90)	33,680	6343	18.83% (18.42, 19.25)	16,716	4522	27.05% (26.38, 27.73)	4,508	1479	32.81% (31.44, 34.20)
80 +	1,563	288	18.43% (16.53, 20.44)	1,355	316	23.32% (21.09, 25.67)	703	201	28.59% (25.28, 32.09)	193	60	31.09% (24.64, 38.13)
**Charlson comorbidity index**												
0	104,530	5369	5.14% (5.00, 5.27)	89,081	7366	8.27% (8.09, 8.45)	45,505	5837	12.83% (12.52, 13.14)	13,362	2148	16.08% (15.46, 16.71)
1 - 15	29,475	2467	8.37% (8.06, 8.69)	24,540	3228	13.15% (12.73, 13.58)	11,371	2207	19.41% (18.69, 20.15)	2,884	701	24.31% (22.75, 25.91)
16 - 30	1,102	138	12.52% (10.63, 14.62)	898	180	20.04% (17.47, 22.82)	391	101	25.83% (21.56, 30.47)	101	30	29.70% (21.02, 39.61)
31 - 50	90	10	11.11% (5.46, 19.49)	73	13	17.81% (9.84, 28.53)	-	-	-	-	-	-
**Index of multiple deprivation (quintiles)**												
1	31,054	1846	5.94% (5.68, 6.21)	26,546	2405	9.06% (8.72, 9.41)	13,422	1835	13.67% (13.09, 14.26)	3,878	629	16.22% (15.07, 17.42)
2	30,218	1799	5.95% (5.69, 6.23)	25,638	2409	9.40% (9.04, 9.76)	12,819	1779	13.88% (13.28, 14.49)	3,474	617	17.76% (16.50, 19.07)
3	27,974	1737	6.21% (5.93, 6.50)	23,721	2324	9.80% (9.42, 10.18)	11,833	1772	14.98% (14.34, 15.63)	3,374	635	18.82% (17.51, 20.18)
4	23,312	1387	5.95% (5.65, 6.26)	19,702	1913	9.71% (9.30, 10.13)	9,771	1420	14.53% (13.84, 15.25)	2,762	496	17.96% (16.54, 19.44)
5	20,591	1104	5.36% (5.06, 5.68)	17,194	1588	9.24% (8.81, 9.68)	8,420	1197	14.22% (13.48, 14.98)	2,451	444	18.12% (16.61, 19.70)
**Rurality**												
Urban	102,665	6004	5.85% (5.71, 5.99)	86,807	8135	9.37% (9.18, 9.57)	43,287	6148	14.20% (13.88, 14.54)	2,154	12,242	17.60% (16.92, 18.28)
Rural	31,760	1944	6.12% (5.86, 6.39)	27,127	2613	9.63% (9.28, 9.99)	13,739	1980	14.41% (13.83, 15.01)	713	3,987	17.88% (16.70, 19.11)
**Ethnicity**												
White	122,261	7672	6.28% (6.14, 6.41)	103,979	10366	9.97% (9.79, 10.15)	52,267	7834	14.99% (14.68, 15.30)	14,908	2,750	18.45% (17.83, 19.08)
Mixed	750	21	2.80% (1.74, 4.25)	609	32	5.25% (3.62, 7.34)	278	22	7.91% (5.03, 11.74)	76	8	10.53% (4.66, 19.69)
Asian	3,722	130	3.49% (2.93, 4.13)	3,088	186	6.02% (5.21, 6.92)	1,465	167	11.40% (9.82, 13.14)	362	75	20.72% (16.66, 25.26)
Black	1,770	27	1.53% (1.01, 2.21)	1,466	53	3.62% (2.72, 4.70)	677	38	5.61% (4.00, 7.62)	171	15	8.77% (4.99, 14.06)
Other	645	15	2.33% (1.31, 3.81)	518	18	3.47% (2.07, 5.44)	250	21	8.40% (5.27, 12.55)	75	9	12.00% (5.64, 21.56)
**Concurrent procedures**												
None	57,208	2686	4.70% (4.52, 4.87)	50,256	3754	7.47% (7.24, 7.70)	28,578	3252	11.38% (11.01, 11.75)	9,370	1,389	14.82% (14.11, 15.56)
Meniscal	77,989	5298	6.79% (6.62, 6.97)	64,336	7033	10.93% (10.69, 11.18)	28,689	4893	17.06% (16.62, 17.50)	6,977	1,490	21.36% (20.40, 22.34)

** = excluding those patients where the date of their procedure was less than this number of years from the end of the observation period in the dataset; - = suppressed due to small numbers; CI = confidence interval; TKA = total or partial knee arthroplasty*

For peer review only

Table 3: Unadjusted and adjusted* risk of knee arthroplasty following arthroscopic chondroplasty

	Unadjusted Risk Subsequent TKA		Adjusted Risk Subsequent TKA	
	**HR**	**95% CI**	**HR**	**95% CI**
**Sex**				
Male	1.00	1.00	1.00	1.00
Female	1.61	1.57, 1.66	1.38	1.34, 1.42
**Age (per five years) ‡**				
Per year	1.35	1.35, 1.36	1.33	1.32, 1.34
**Year of treatment (per five years)**				
Year	0.99	0.96, 1.03	0.95	0.92, 0.98
**Charlson comorbidity index (per five units)**				
Charlson index	1.29	1.27, 1.31	1.03	1.01, 1.05
**Index of multiple deprivation (quintile)**				
1 = least	1.00	1.00	1.00	1.00
2	1.03	0.99, 1.08	1.07	1.03, 1.12
3	1.08	1.04, 1.13	1.17	1.12, 1.22
4	1.03	0.99, 1.08	1.20	1.15, 1.26
5 = most	1.01	0.96, 1.06	1.29	1.23, 1.36
**Rurality**				
Urban	1.00	1.00	1.00	1.00
Rural	1.03	1.00, 1.07	0.99	0.95, 1.02
**Ethnicity**				
White	1.00	1.00	1.00	1.00
Mixed	0.50	0.38, 0.65	0.66	0.51, 0.86
Asian	0.65	0.59, 0.72	0.73	0.66, 0.81
Black	0.35	0.28, 0.42	0.44	0.36, 0.54
Other	0.34	0.24, 0.48	0.45	0.32, 0.64
**Concurrent procedures**				
None	1.00	1.00	1.00	1.00
Meniscal surgery	1.52	1.48, 1.57	1.09	1.06, 1.13

** adjusted by all variables in the table; ‡ age <20 years suppressed due to small numbers; HR = hazard ratio; CI = confidence interval; TKA = total or partial knee arthroplasty*

For peer review only

Table 4: Rates and relative risk of undergoing TKA with previous chondroplasty by age at TKA in 2016-17

	**Prior chondroplasty**		**Without prior chondroplasty**		**Relative Risk**	
**Age at TKA (years)**	**Annual Rate TKA /100k**	**95% CI**	**Annual Rate TKA /100k**	**95% CI**	**RR**	**95% CI**
30 - 39	274.48 (0.27%)	190.16, 383.35 (0.19%, 0.38%)	1.60 (0.00%)	1.32, 1.92 (0.00%, 0.00%)	170.92	116.72, 250.30
40 - 49	1454.02 (1.45%)	1318.04, 1600.06 (1.32%, 1.60%)	19.79 (0.02%)	18.79, 20.82 (0.02%, 0.02%)	72.45	65.00, 80.76
50 - 59	3626.62 (3.63%)	3448.20, 3811.60 (3.45%, 3.81%)	130.68 (0.13%)	128.05, 133.35 (0.13%, 0.13%)	26.82	25.41, 28.30
60 - 69	5179.17 (5.18%)	4933.67, 5433.20 (4.93%, 5.43%)	386.68 (0.39%)	381.64, 391.77 (0.38%, 0.39%)	12.78	12.16, 13.44
70 +	6090.50 (6.09%)	5721.53, 6475.82 (5.72%, 6.48%)	520.46 (0.52%)	514.89, 526.07 (0.51%, 0.53%)	11.09	10.42, 11.80
Overall (30 +)	3494.61 (3.49%)	3394.82, 3596.52 (3.39%, 3.60%)	195.38 (0.20%)	193.90, 196.87 (0.19%, 0.20%)	17.32	16.81, 17.84

TKA = total or partial knee arthroplasty; RR = risk ratio

For peer review only

FIGURES

Figure 1: Flow chart illustrating extraction of patient level cohort

For peer review only

Figure 2: Survival curve (not undergoing knee arthroplasty) following chondroplasty by age†

† Age groups < 20 years and 80+ years suppressed due to small numbers

For peer review only

Figure 3: Survival curve (not undergoing knee arthroplasty) following chondroplasty by sex*

For peer review only

** Age groups < 20 years and 80+ years suppressed due to small numbers*

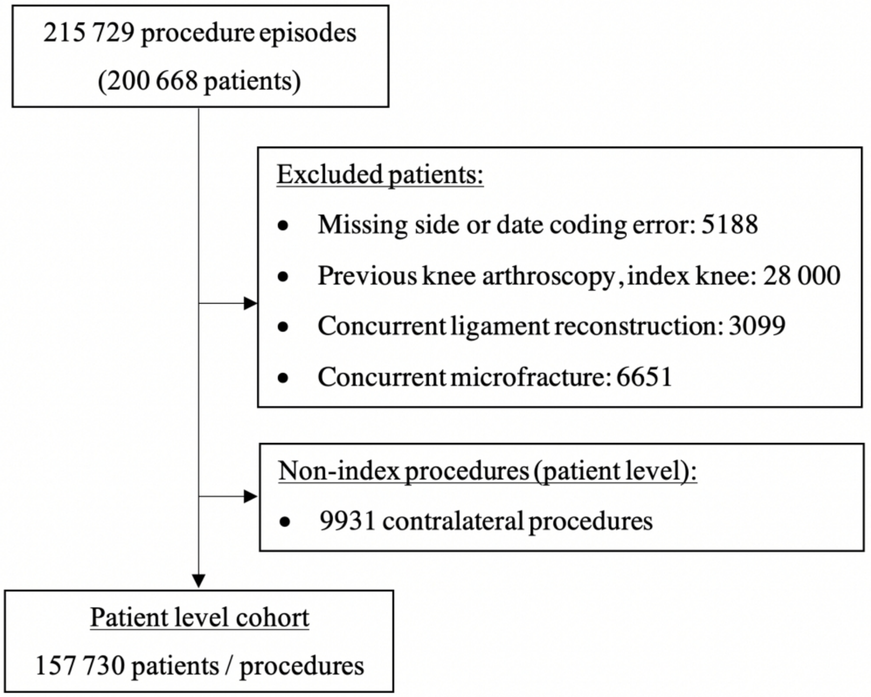

Flow chart illustrating extraction of patient level cohort

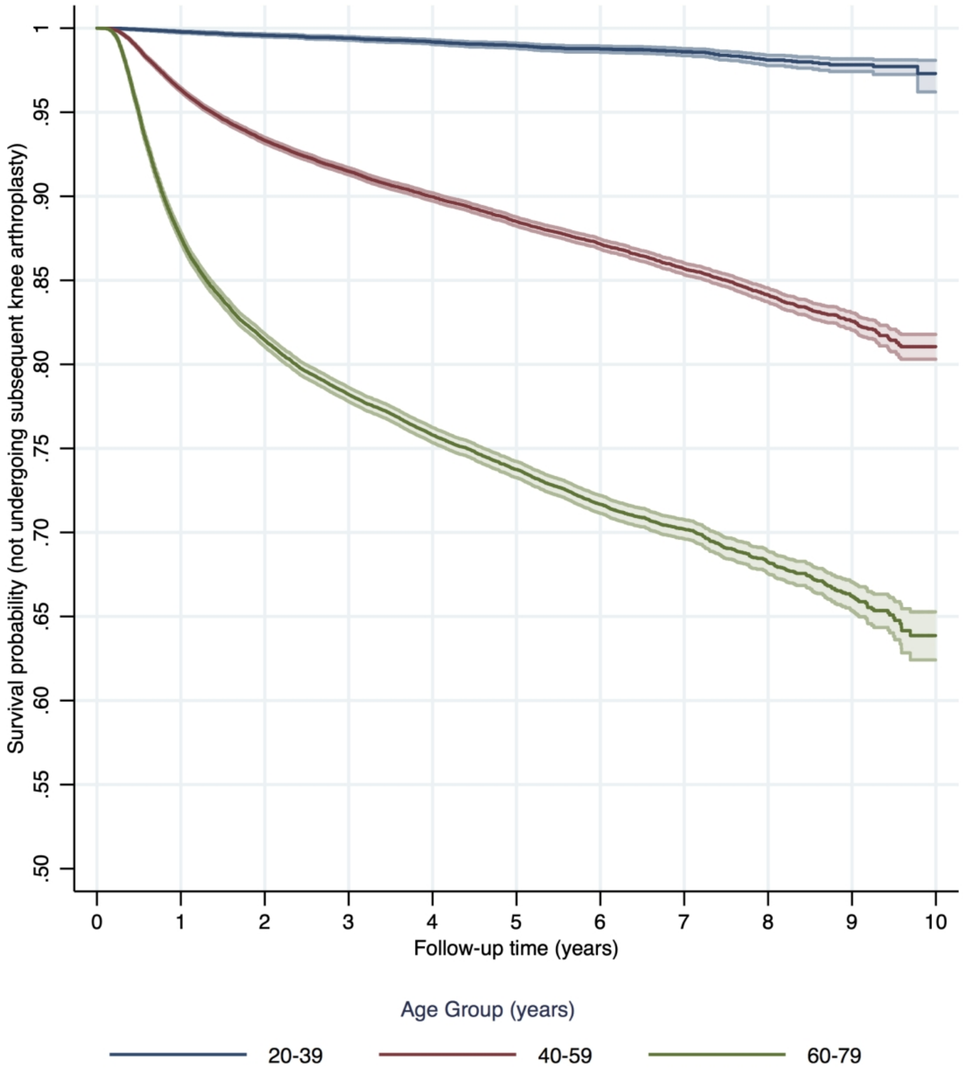

† Age groups < 20 years and 80+ years suppressed due to small numbers

Figure 2: Survival curve (not undergoing knee arthroplasty) following chondroplasty by age†

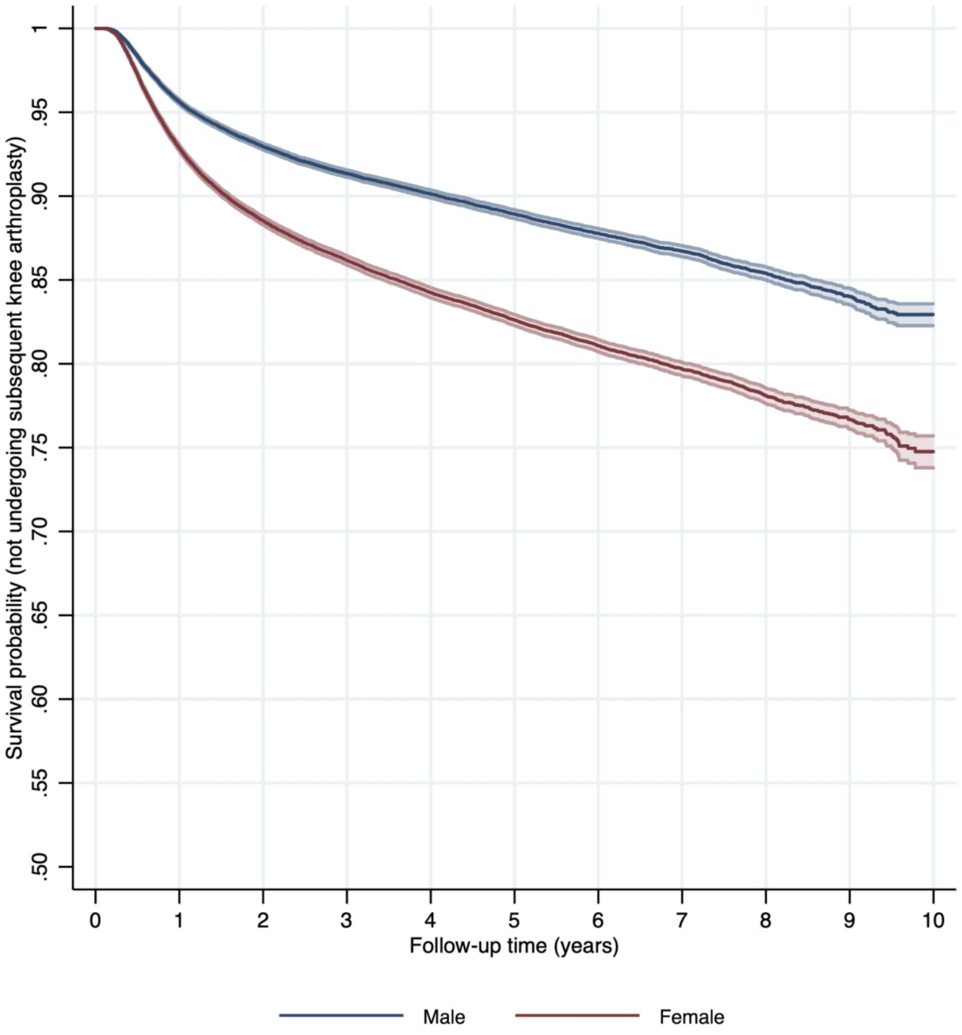

** Age groups < 20 years and 80+ years suppressed due to small numbers*

Figure 3: Survival curve (not undergoing knee arthroplasty) following chondroplasty by sex*

Appendix 1: OPCS procedure code list

PROCEDURE	OPCS 4.4	OPCS 4.5	OPCS 4.6	OPCS 4.7	Description
Chondroplasty*	W83.3*	W83.3*	W83.3*	W83.3*	Endoscopic shaving of articular cartilage
Chondroplasty*	W83.4*	W83.4*	W83.4*	W83.4*	Endoscopic articular abrasion chondroplasty
Chondroplasty*	W83.5*	W83.5*	W83.5*	W83.5*	Endoscopic articular thermal chondroplasty
Chondroplasty*	W83.6*	W83.6*	W83.6*	W83.6*	Endoscopic excision of articular cartilage NEC
Chondroplasty*	W89.1*	W89.1*	W89.1*	W89.1*	Endoscopic chondroplasty NEC
Microfracture*	W83.1*	W83.1*	W83.1*	W83.1*	Endoscopic drilling of lesion of articular cartilage
Microfracture*	W84.5*	W84.5*	W84.5*	W84.5*	Endoscopic drilling of epiphysis for repair of articular cartilage
Meniscal surgery	W82.2	W82.2	W82.2	W82.2	Endoscopic resection of semilunar cartilage NEC
Meniscal surgery	W82.3	W82.3	W82.3	W82.3	Endoscopic repair of semilunar cartilage
Meniscal surgery	W82.1	W82.1	W82.1	W82.1	Endoscopic total excision of semilunar cartilage
Ligament reconstruction (exclusion)*	W74.2	W74.2	W74.2	W74.2	Reconstruction of intra-articular ligament NEC
Ligament reconstruction (exclusion)*	W84.1	W84.1	W84.1	W84.1	Endoscopic repair of intra-articular ligament
Ligament reconstruction (exclusion)*	W84.2	W84.2	W84.2	W84.2	Endoscopic reattachment of intra-articular ligament
Ligament reconstruction (exclusion)*	W72.3	W72.3	W72.3	W72.3	Primary prosthetic replacement of intra-articular ligament
Ligament reconstruction (exclusion)*	W72.4	W72.4	W72.4	W72.4	Prosthetic replacement of intra-articular ligament NEC
Arthroplasty*	W43.1	W43.1	W43.1	W43.1	Primary total prosthetic replacement of joint using cement NEC
Arthroplasty*	W44.1	W44.1	W44.1	W44.1	Primary total prosthetic replacement of joint not using cement NEC
Arthroplasty*	W44.8	W44.8	W44.8	W44.8	Other specified total prosthetic replacement of other joint not using cement
Arthroplasty*	W44.9	W44.9	W44.9	W44.9	Unspecified total prosthetic replacement of other joint not using cement
Arthroplasty*	W45.1	W45.1	W45.1	W45.1	Primary total prosthetic replacement of joint NEC
Arthroplasty*	W45.8	W45.8	W45.8	W45.8	Other specified other total prosthetic replacement of other joint
Arthroplasty*	W45.9	W45.9	W45.9	W45.9	Unspecified other total prosthetic replacement of other joint
Arthroplasty*	W53.8	W53.8	W53.8	W53.8	Other specified prosthetic replacement of articulation of other bone not using cement
Arthroplasty	W40.1	O18.1	O18.1	O18.1	Primary hybrid prosthetic replacement of knee joint using cement
Arthroplasty	W40.8	O18.8	O18.8	O18.8	Other specified hybrid prosthetic replacement of knee joint using cement
Arthroplasty	W40.9	O18.9	O18.9	O18.9	Unspecified hybrid prosthetic replacement of knee joint using cement
Arthroplasty	W40.1	W40.1	W40.1	W40.1	Primary total prosthetic replacement of knee joint using cement
Arthroplasty	W40.8	W40.8	W40.8	W40.8	Other specified total prosthetic replacement of knee joint using cement
Arthroplasty	W40.9	W40.9	W40.9	W40.9	Unspecified total prosthetic replacement of knee joint using cement
Arthroplasty	W41.1	W41.1	W41.1	W41.1	Primary total prosthetic replacement of knee joint not using cement
Arthroplasty	W41.8	W41.8	W41.8	W41.8	Other specified total prosthetic replacement of knee joint not using cement
Arthroplasty	W41.9	W41.9	W41.9	W41.9	Unspecified total prosthetic replacement of knee joint not using cement
Arthroplasty	W42.1	W42.1	W42.1	W42.1	Primary total prosthetic replacement of knee joint NEC
Arthroplasty	W42.8	W42.8	W42.8	W42.8	Other specified other total prosthetic replacement of knee joint
Arthroplasty	W42.9	W42.9	W42.9	W42.9	Unspecified other total prosthetic replacement of knee joint
Arthroplasty*	W58.1	W58.1	W58.1	W58.1	Primary resurfacing arthroplasty of joint

**: Additional site-specific knee code required (Z846, Z765, Z845, Z844, Z774, or Z787)*

For peer review only

Appendix 2: Flow chart illustrating the extraction of the knee arthroplasty cohort

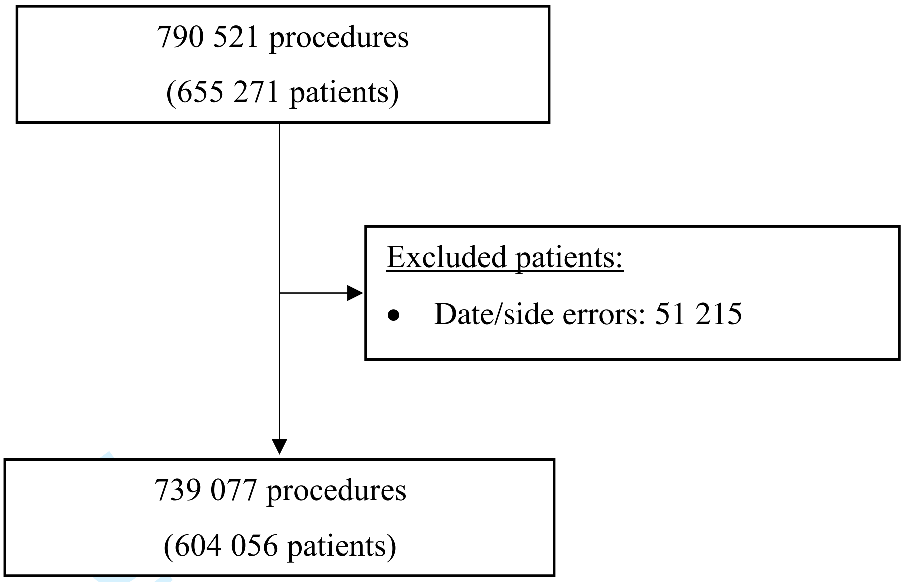

For peer review only
